# Predicting rejection of emerging contaminants through RO membrane filtration based on ANN-QSAR modeling approach: trends in molecular descriptors and structures towards rejections†

**DOI:** 10.1039/d3ra03177b

**Published:** 2023-08-08

**Authors:** Setare Loh Mousavi, S. Maryam Sajjadi

**Affiliations:** a Faculty of Chemistry, Semnan University Semnan Iran sajjadi@semnan.ac.ir +98 23 33384110 +98 23 31533192

## Abstract

In this work, a quantitative structure–activity relationship (QSAR) study was performed on a set of emerging contaminants (ECs) to predict their rejections by reverse osmosis membrane (RO). A wide range of molecular descriptors was calculated by Dragon software for 72 ECs. The QSAR data was analyzed by an artificial neural network method (ANN), in which four out of 3000 theoretical molecular descriptors were chosen and their significance was computed based on the Garson method. The significance trends of descriptors were as follows in descending order: ESpm14u > R2e > SIC1 > EEig03d. The selected descriptors were ranked based on their importance and then an explorative study was conducted on the QSAR data to show the trends in molecular descriptors and structures toward the rejections values of ECs. The MLR algorithm was used to make a linear model and the results were compared with those of the nonlinear ANN algorithm. The comparison results revealed it is necessary to apply the ANN model to this data with non-linear properties. For the whole dataset, the correlation coefficient (*R*^2^) and residual mean squared error (RMSE) of the ANN and MLR methods were 0.9528, 6.4224; and 0.8753, 11.3400, respectively. The comparison results showed the superiority of ANN modeling in the analysis of ECs' QSAR data.

## Abstract 2

QSAR-ANN modelling was applied on ECs to predict the rejection of ECs by RO membrane and conduct explanatory study based the importance of selected descriptors.

## Graphical abstract

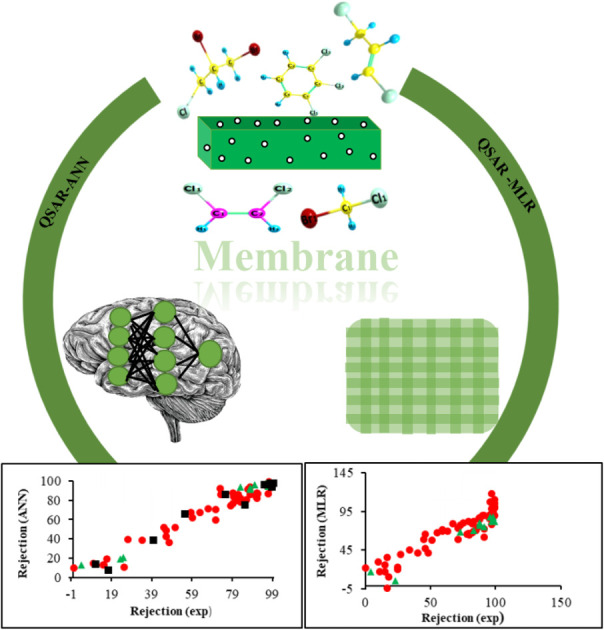

## 1. Introduction

In recent decades, the excess rise in water demand occurred due to the increased population and industrial and agricultural expansion, which may be satisfied by avoiding pollution of freshwater supplies and developing wastewater treatment strategies. In the early years of the 1800's, newly identified compounds of anthropogenic were discovered in the aquatic environment and other water resources, becoming a global issue of increasing environmental concern. Later, these contaminations were referred to as emerging contaminants (ECs).^1,2^ ECs are commonly organic in nature and typically exist at low concentrations in the range ng. L^−1^ to μg L^−1^.^3–5^ The ECs can be carcinogenic to vital organs of the human body and can cause unpleasant taste and odor to the water.^6^ Consequently, the removal of them from drinking water is greatly significant. Conventional wastewater treatment processes (WWTPs) are the standard strategies to remove a variety kind of contaminates such as suspended and colloidal particulates, nutrients, and pathogens from wastewater; however, they are not led to efficient removal of the ECs.^7,8^ Most of the ECs are often associated with discharges from WWTPs because of the universal usage of many of these compounds and a lack of strategies with appropriate removal efficiency, such as adsorption, oxidation processes, and their combinations.^7^ Moreover, several techniques have been applied to remove ECs during the last several decades, including biological methods and advanced processes.^9,10^

Biological treatment strategies include two types of processes such as aerobic and anaerobic. Some common aerobic technologies are membrane bioreactors, active sludge, and a sequencing batch reactor. Anaerobic treatments include anaerobic film reactors and anaerobic sludge reactors.^10,11^ However, biological and conventional wastewater treatment display limited performance. For instance, they are not able enough to entirely remove certain ECs to acceptable concentration levels in which they are safe for human utilization. Overall, biological processes and conventional treatment strategies are not versatile toward the removal of different classes of micropollutants and they lead to insufficient removal of many micropollutants from water.^12–14^

On the contrary, advanced processes have shown great ability to degrade or remove many of these ECs.^15^ There are many advanced technologies like ultraviolet light, activated carbon, and membrane.^16,17^ The membrane filtration process includes nanofiltration (NF), microfiltration (MF), ultrafiltration (UF), and reverse osmosis (RO) methods. One of the most important membrane filtrations is the RO membrane which processes the solution–diffusion mechanism for transporting organic solutes over the osmotic membranes.^18^

Although RO membrane can provide efficient removal of various high molecular weight (MW) compounds such as pharmaceuticals, this is inefficient for the removal of low MW compounds. The permeation of organic molecules on RO membranes can be affected by three important factors: (i) RO operating conditions such as temperature, and pH; (ii) membrane properties, for instance, membrane fouling, and pressure; and (iii) molecular physicochemical properties of contaminants including charge/shape/size, functional groups, and hydrophobicity.^19,20^ In determining the rejection of compounds by membranes, a crucial challenge is membrane fouling. Although membrane cleaning can reverse fouling and as a result prolong its useful lifespan, it needs chemicals that may degrade the structure of membranes. The difficulties in operational experiments guide researchers to find an ideal model to correlate the structures of ECs and their rejections which can apply to predicting the rejection of a wide range of new ECs.^16,20^

Quantitative structure–activity relationship (QSAR) is an efficient developed model in computational chemistry and used in different scientific fields (environmental engineering, material science, toxicology, and medicinal chemistry) for correlating, quantitatively an activity or property of molecules with chemical structures.^21,22^ This method finds the relationship between the molecular structure and its physicochemical properties to evaluate the structure and properties of new molecules without experimenting.^23^

In the QSAR method, theoretical descriptors are a group of numerical indices that are associated with the structure of molecules and encode information about the structure.^24–26^ There is a variety type of descriptors such as the number of walks and paths, topological descriptors, three-dimensional MoRSE descriptors, standing for molecular representation of structures based on Electronic diffraction; and counting of functional groups.^27–30^

There are various software for computing descriptors, some of which commonly used are as follows: comparative receptor surface analysis (CoRSA), comparative molecular field analysis (CoMFA), self-organizing molecular field analysis (SOMFA), hydrophobic interactions (HINT), property evaluation by class variables (PRECLAV), and Dragon.^31–36^

Each software possesses a different algorithm and provides different kinds of descriptors. CoMFA is based on molecular field analysis and represents real three-dimensional descriptors.^37^ CoRSA generates a virtual receptor model by considering the common electrostatic and steric properties of a set of molecules.^31^ SOMFA has a similarity in concept with CoMFA and can be applied in three-dimensional QSAR studies.^38^ HINT has been designed to map and calculate the hydrophobic environment of small proteins and molecules.^32^ The PRECLAV computes almost 400 constitutional, geometrical, topological, electrostatic, electronic, and quantum “global” descriptors.^39^ The Dragon can provide nearly 5000 molecular descriptors composed of not only the simplest atom types, fragment counts, and functional groups, but also several geometrical and topological.^40^

The predictive capability of the QSAR technique is determined by the method used for modeling. Two methods of linear and non-linear modeling determine the mathematical modeling between descriptors and their molecular activity. Linear methods consist of stepwise regression, principal component regression (PCR), principal component analysis (PCA), kernel stone, multiple linear regression (MLR), particle least squares (PLS); and nonlinear approaches including support vector machine (SVM), Kohonen self-organizing map (SOM), radial basis function (RBF), and artificial neural networks (ANN).^41–47^ The ANN algorithms are non-linear models that make a mapping of the input and output variables, in turn, the map is utilized to predict unknown output as a function of appropriate descriptors.^48^ The main advantage of ANN methods is that they can incorporate and combine both experimental data and literature-based to solve many problems such as predicting membrane permeability and membrane rejection. This predictive power can be captured to virtually analyze the properties of molecules before testing them in a laboratory.^44,45,49–52^

There are some publications on applying QSAR modeling to predict the rejection of pollutants using different modeling strategies.^16,17,29,50,53,54^ For instance, Yangali-Quintanilla *et al.* studied the rejection of ECs by NF membranes. They used PLS and MLR algorithms on QSAR rejection data to find the relationship between filtration operating conditions, membrane properties, compound properties; and rejection of molecules. Although they applied PCA and stepwise to reduce the number of variables in the modeling processes, the obtained *R*^2^ from modeling approaches was up to 0.84. The small value of *R*^2^ could be because of the presence of nonlinearity in the data.^55^ In another research, Yangali-Quintanilla *et al.* investigated the rejection of molecules using QSAR data and ANN modeling.^56^ They applied PCA on QSAR data to diminish the number of input variables however, PCA suffers the risk of selecting variables from the input space that may not be related to the output variable of MLR. Moreover, the authors did not examine the importance of the selected descriptors and they did not interpret the trend of ECs' rejections according to theoretical descriptors. Indeed, to the best of our knowledge, there is no exploration study on the relationship between the theoretical molecular descriptors and structure properties of contaminants in their rejection by RO membrane.

Here, we use the experimental data set reported by Breitner *et al.* to address these neglected issues.^57^ The variable selection was conducted on QSAR data based on the correlation between the descriptors and rejections. The chosen descriptors were those having high correlation with response and less correlation with the another descriptors.

In this study, we have two main goals; the first one is developing an ANN-QSAR modeling approach for the prediction of rejection compounds according to their structural characteristics by RO membrane. The second one is investigating the effect of functional groups on chemical properties and finding the interactions between compounds and membranes. The interactions depend on some factors such as hydrophobicity/hydrophilicity of molecules and electronegativity of their functional groups, molecular size, and polarity.^57^

In this work, ANN analysis is applied to QSAR data of ECs using four selected theoretical descriptors including structural information content index (neighborhood symmetry of 1-order) abbreviated as SIC1, R autocorrelation of lag 2/weighted by Sanderson electronegativity (R2e), eigenvalue 03 from edge adjacency matrix weighted by dipole moment (EEig03d), and spectral moment 14 from edge adjacency matrix (ESpm14u). A comprehensive study is conducted on interpreting the QSAR data to understand the relationship between molecular structures of ECs and their rejections based on the values of the selected theoretical descriptors.

## 2. Materials and methods

### 2.1 Molecular database

This study utilized a data set comprised of 72 ECs molecules and the rejection percentage of each molecule, henceforth called rejection for simplicity.^57,58^ The membrane used is the Hydranautics ESPA2-LD. The ECs were spiked into the tank containing buffered Deionized water with varied concentrations between 150 μg L^−1^ to 3 mg L^−1^ depending on volatility, detection limits, and the expected rejection of individual pollutants. The average water mass transfer coefficient of the ESPA2-LD was calculated from the experimental data to be 4.50 L m^−2^ h^−1^ bar^−1^, concentration polarization coefficient (*β* = 1.2), and net transmembrane pressure (Δ*P* − Δπ) = 10 bar. Table 1 shows the molecular structures and the rejections of all molecules. Due to the limited space, the standard deviation of the rejections measurements were represented in Table S1.†

**Table 1:** The structure of molecules and rejection of each ECs in QSAR-ANN studies^57,58^

ID	Compound	Abbrev.	Structure	Rejection (ANN)	Rejection (exp)	Set of data
1	1,1,1,2-Tetrachloroethane	1,1,1,2-TCA	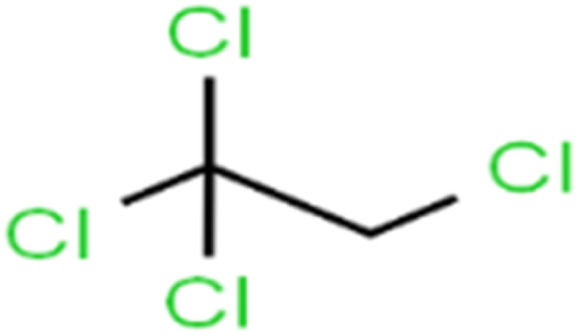	94.1	99	Training
2	1,1,1-Trichloroethane	1,1,1-TCA	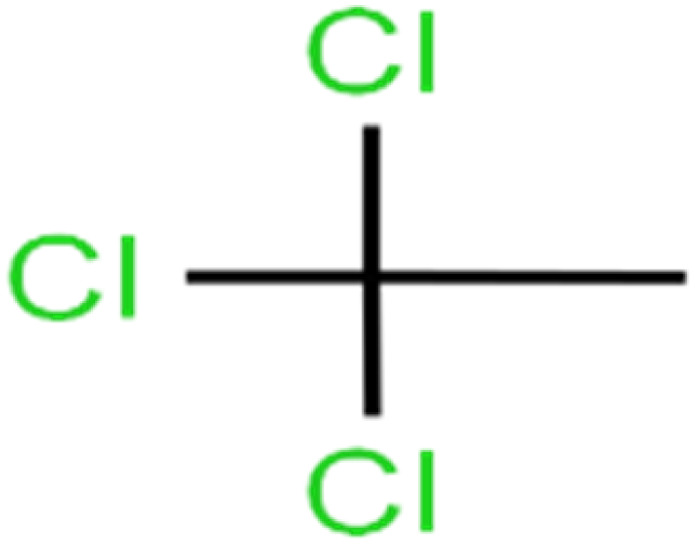	93.1	98	Training
3	1,1,2,2-Tetrachloroethane	1,1,2,2-TCA	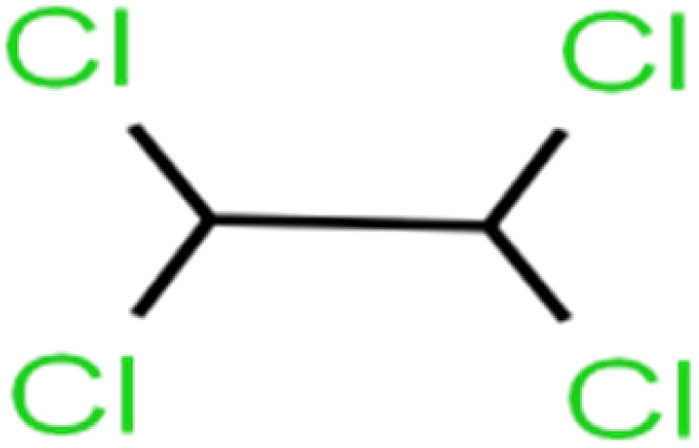	96.6	97	Validation
4	1,1,2-Trichloroethane	1,1,2-TCA	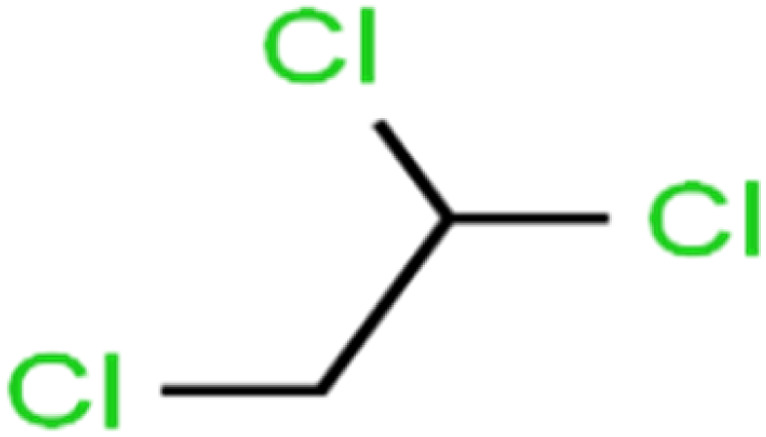	81.1	86	Training
5	1,1-Dichloroethane	1,1-DCA	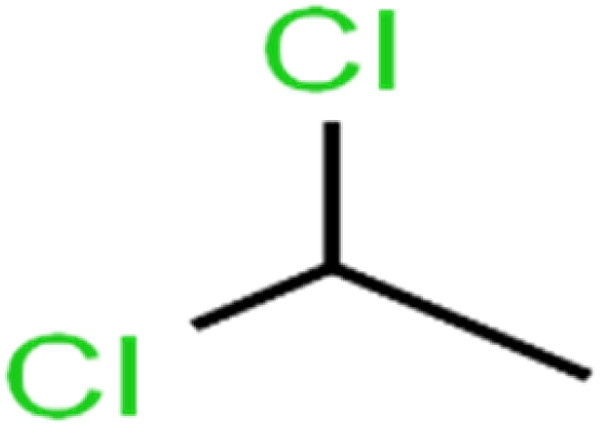	83.7	80	Training
6	1,1-Dichloroethene	1,1-DCE	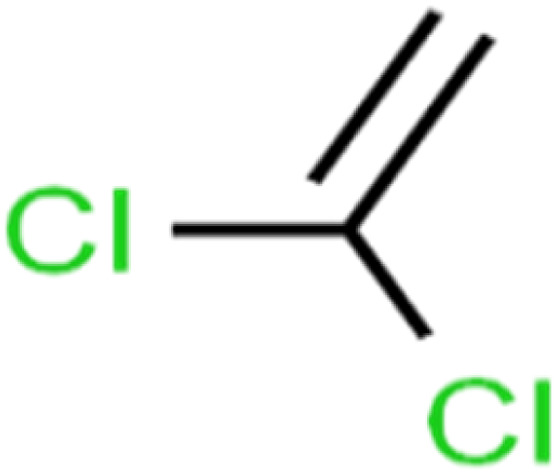	19.2	17	Training
7	1,1-Dichloropropene	1,1-DCP	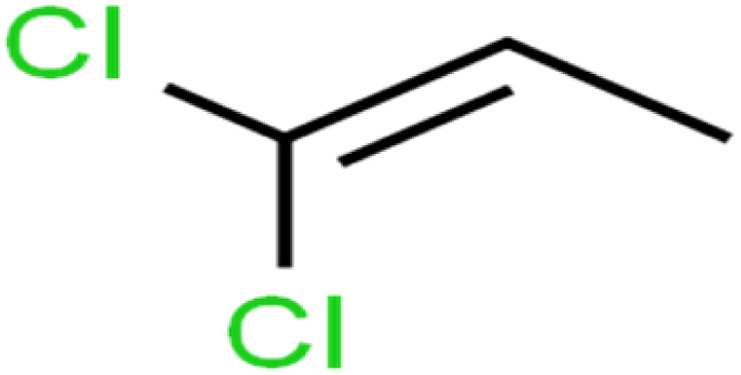	51.9	45	Training
8	1,2,3-Trichlorobenzene	1,2,3-TCB	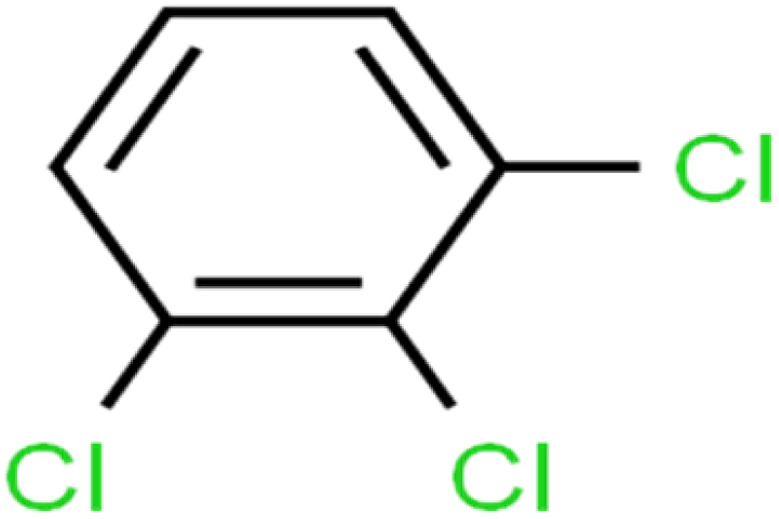	87.2	91	Validation
9	1,2,3-Trichloropropane	1,2,3-TCP	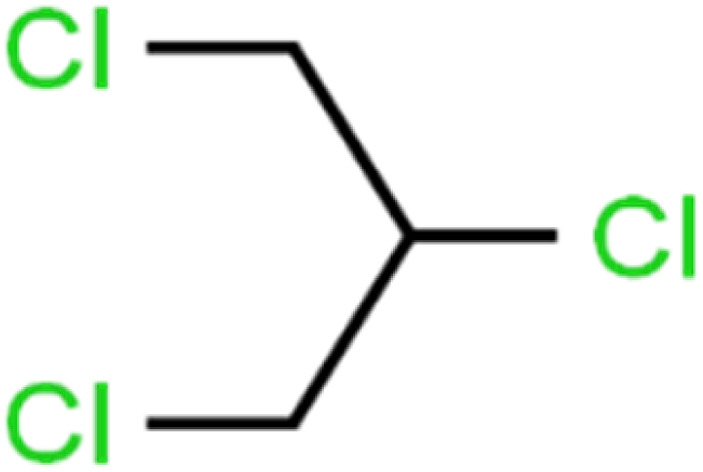	96.4	95	Test
10	1,2,4-Trichlorobenzene	1,2,4-TCB	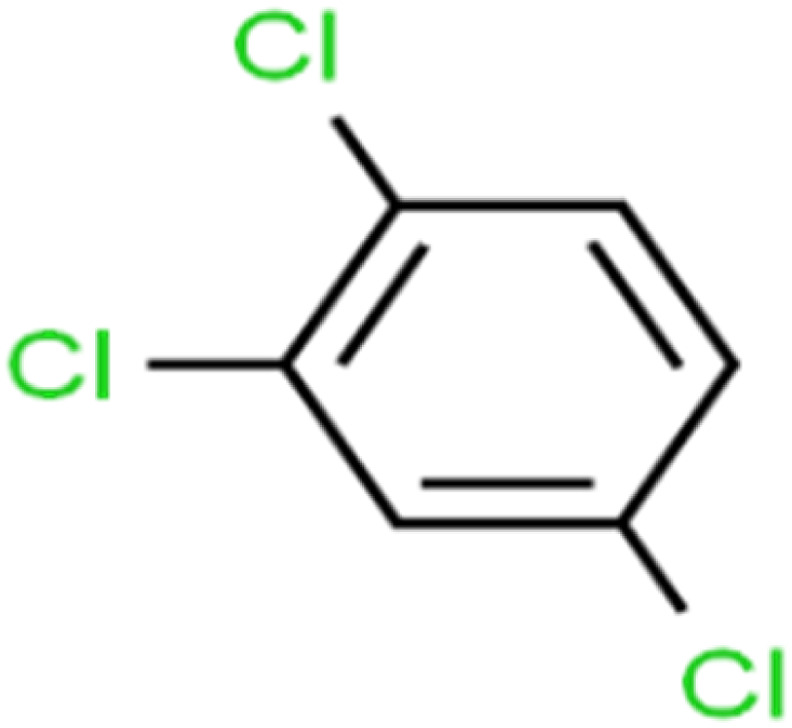	88.0	79	Training
11	1,2,4-Trimethylbenzene	1,2,4-TMB	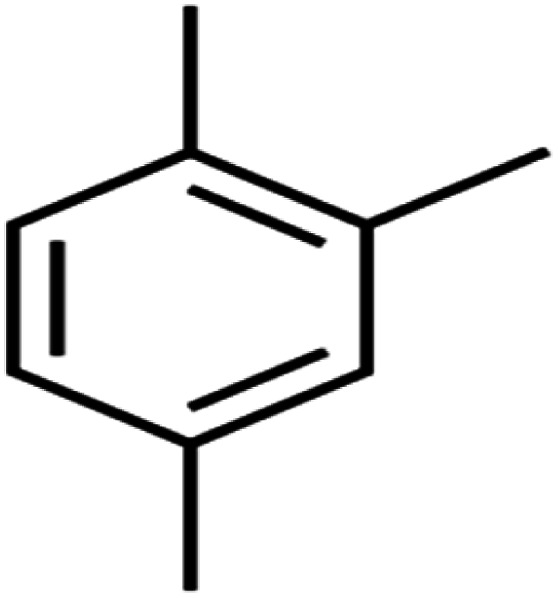	97.6	97	Training
12	1,2-Dibromo-3-chloropropane	1,2-DB-3-CP	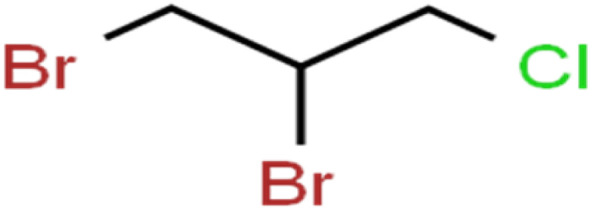	87.1	97	Training
13	1,2-Dibromoethane	EDB	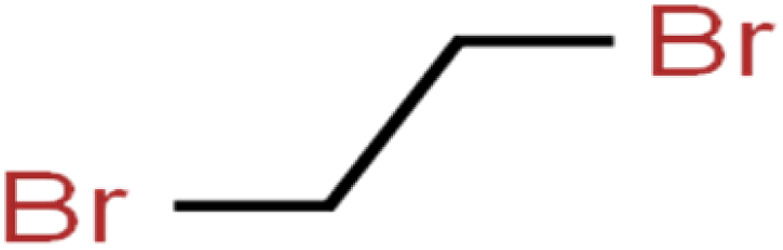	39.1	40	Test
14	1,2-Dichlorobenzene	1,2-DCB	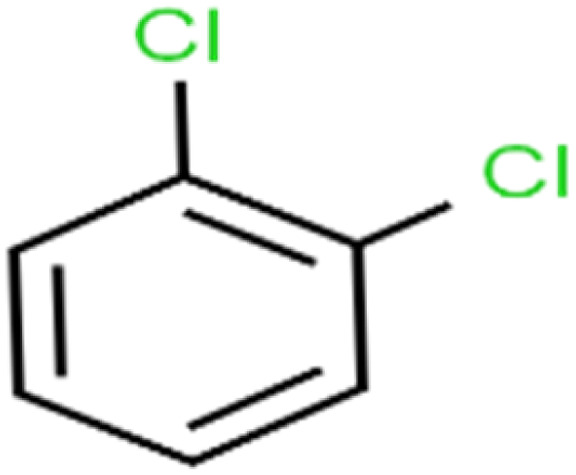	78.9	83	Validation
15	1,2-Dichloroethane	1,2-DCA	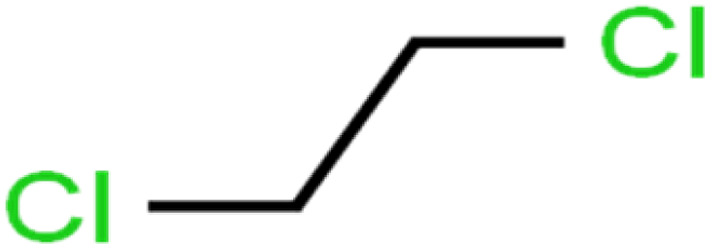	38.2	34	Training
16	1,2-Dichloropropane	1,2-DCP	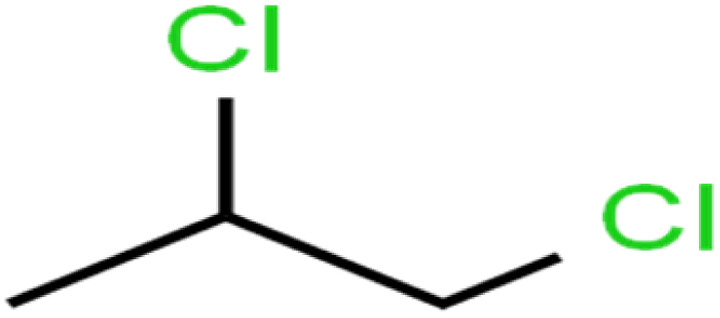	82.6	91	Training
17	1,3,5-Trimethylbenzene	1,3,5-TMB	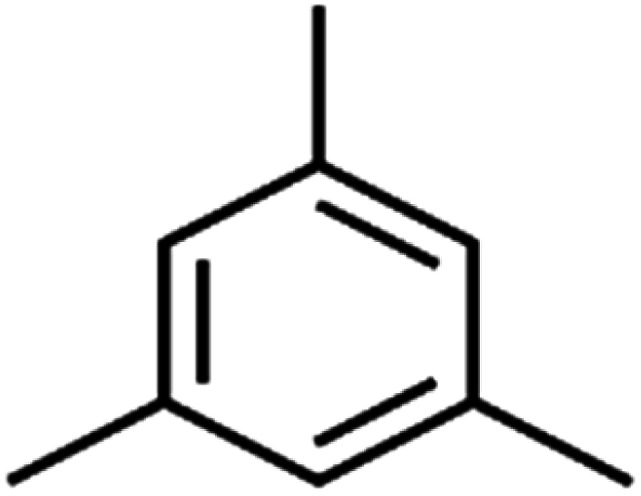	89.2	99	Training
18	1,3-Dichlorobenzene	1,3-DCB	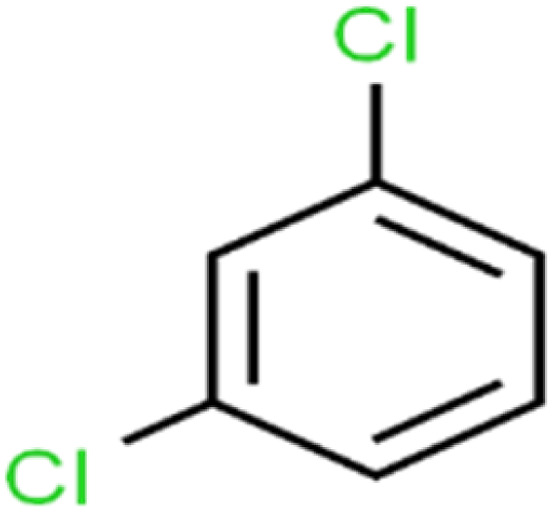	70.0	71	Training
19	1,3-Dichloropropane	1,3-DCP	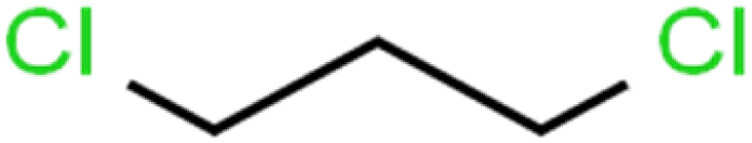	60.2	71	Training
20	1,4-Dichlorobenzene	1,4-DCB	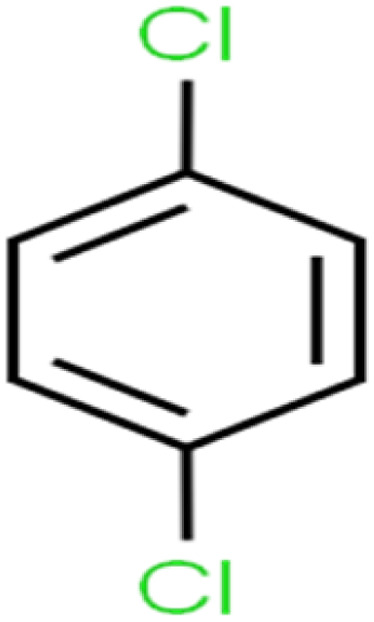	62.5	59	Training
21	1,4-Dioxane	1,4-D	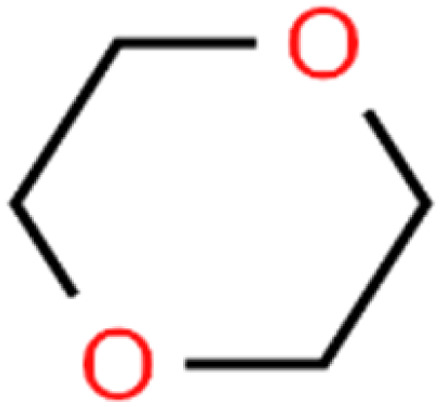	98.5	98	Training
22	2-Butanone	2-But	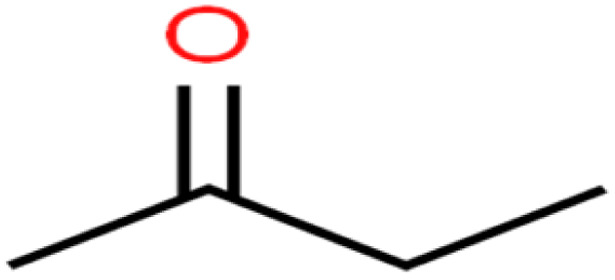	86.1	73	Training
23	2-Chlorotoluene	2-CT	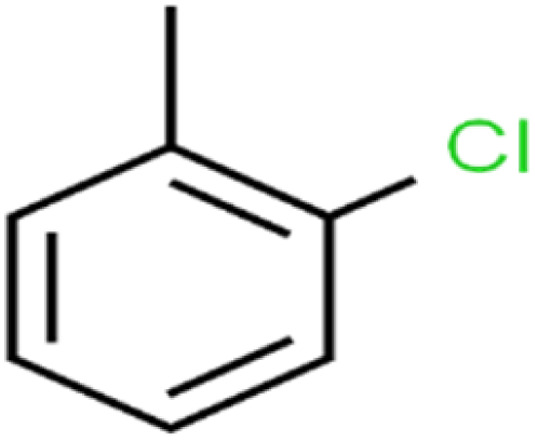	86.7	88	Test
24	2-Hexanone	2-Hex	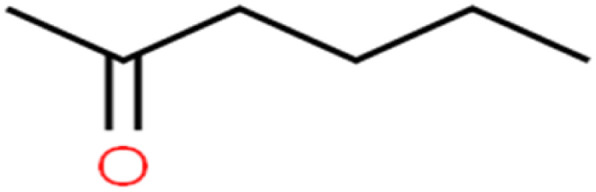	93.8	83	Training
25	4-Chlorotoluene	4-CT	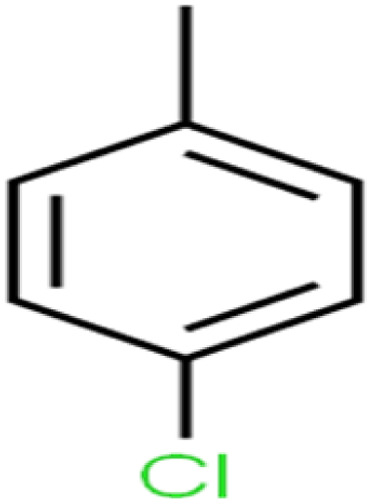	71.0	67	Training
26	4-Isopropyltoluene	4-IPT	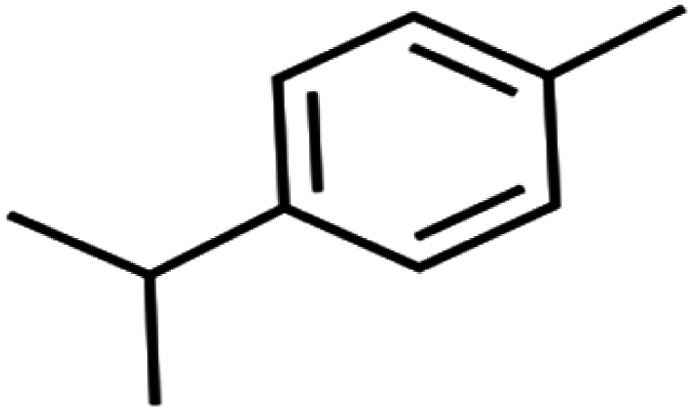	96.6	98	Validation
27	4-Methyl-2-pentanone	MIBK	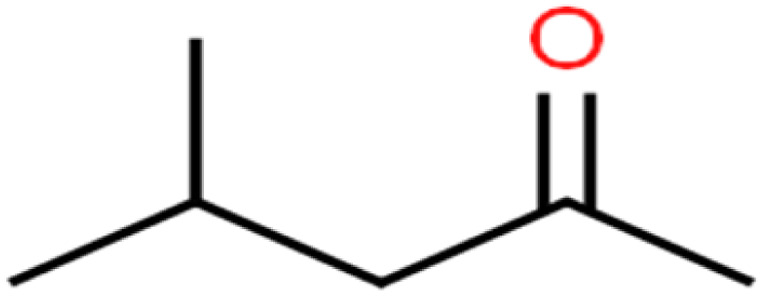	95.9	98	Training
28	Acetone	Acetone	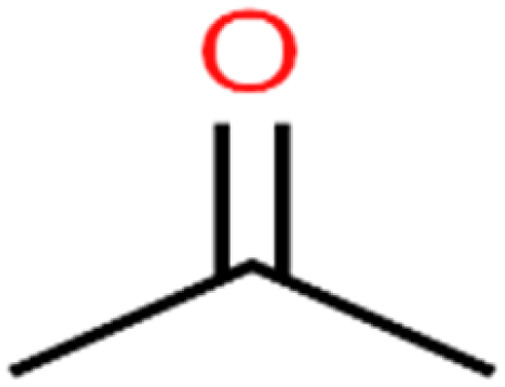	65.2	55	Test
29	Acetonitrile	Ace-N	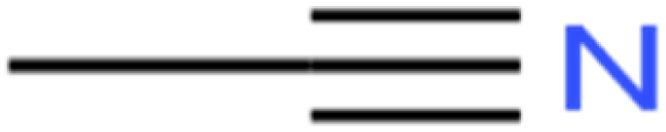	19.1	23	Validation
30	Acrylonitrile	Acr-N	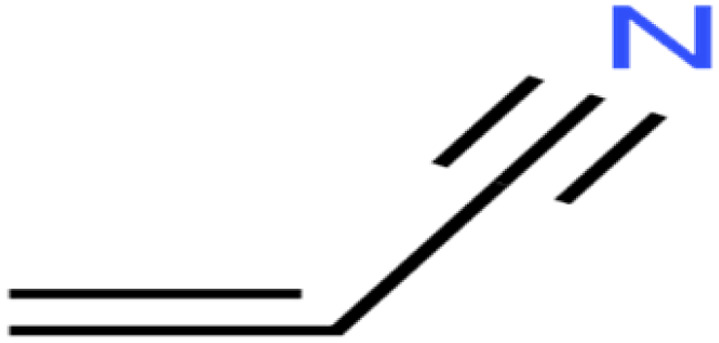	7.5	18	Test
31	Benzene	Benzene	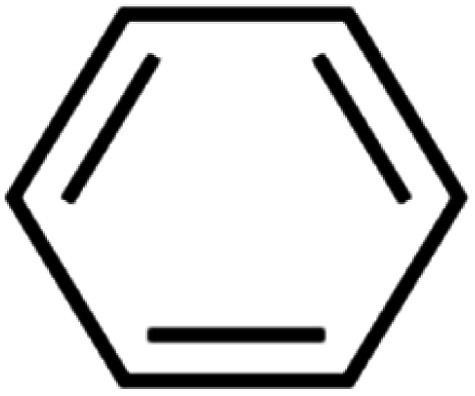	76.3	79	Training
32	Bromobenzene	BB	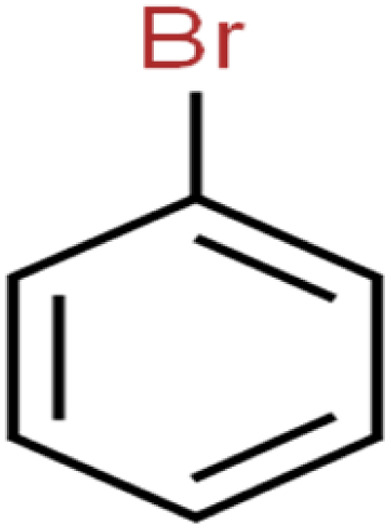	67.1	59	Training
33	Bromochloromethane	BCM	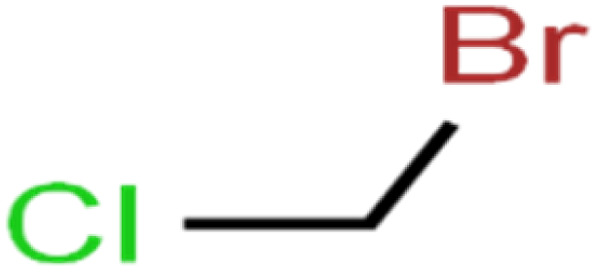	20.2	25	Training
34	Bromodichloromethane	BDCM	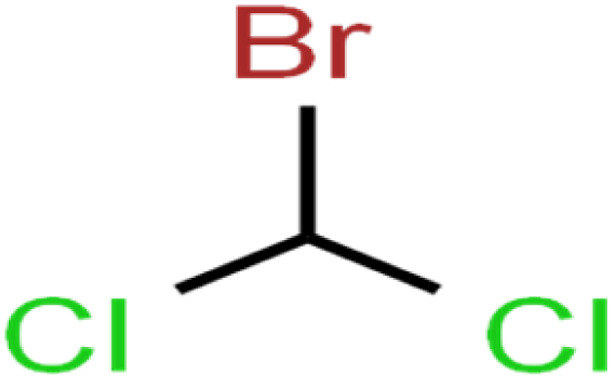	80.4	82	Training
35	Bromoform	BF	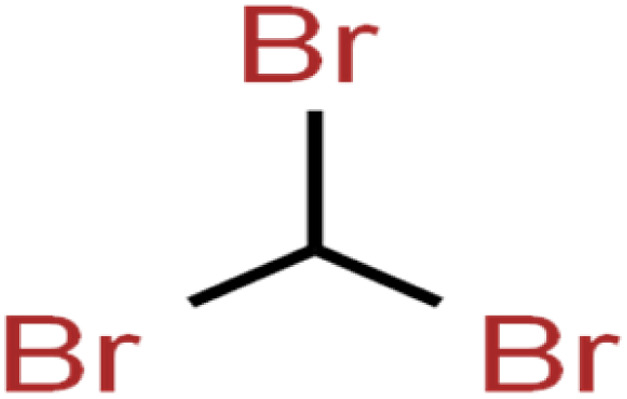	75.0	85	Test
36	Bromomethane	BM	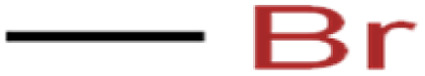	9.7	0	Training
37	Carbon tetrachloride	C-Tet	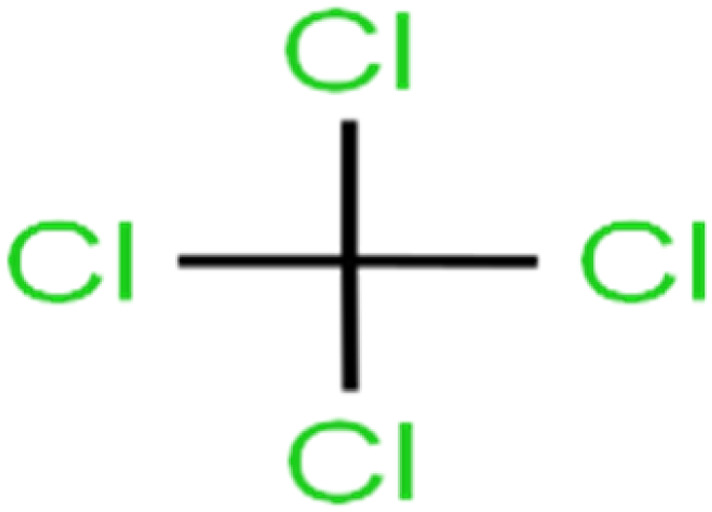	98.8	97	Training
38	Chlorobenzene	CB	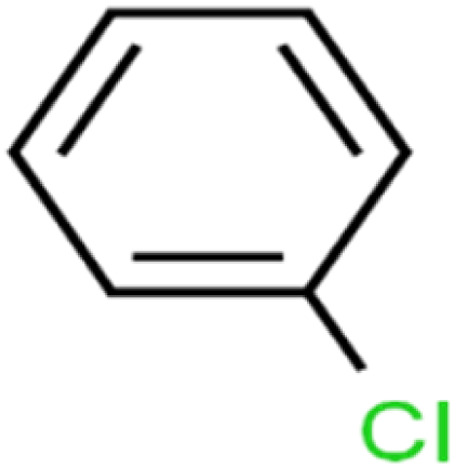	67.5	63	Training
39	Chloroethane	CA	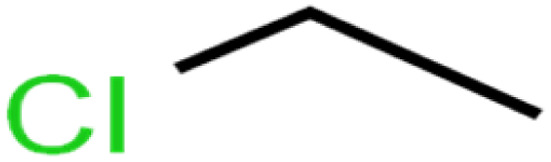	12.8	15	Training
40	Chloroform	CF	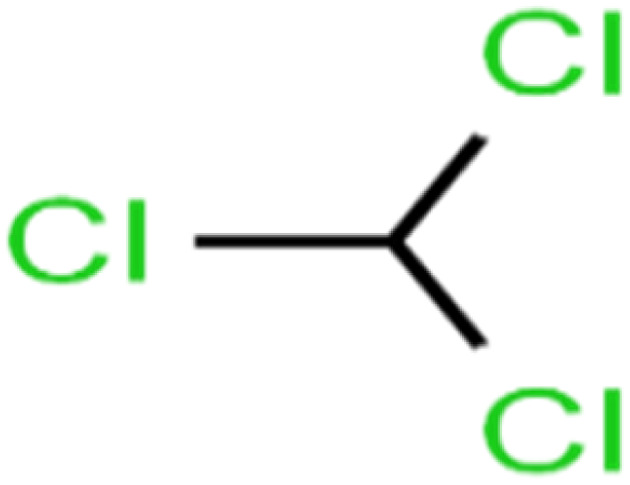	91.9	73	Validation
41	Chloromethane	CM	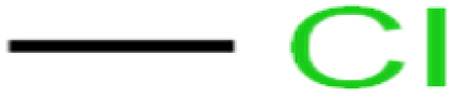	12.8	4	Validation
42	*cis*-1,2-Dichloroethene	*cis*-1,2-DCE	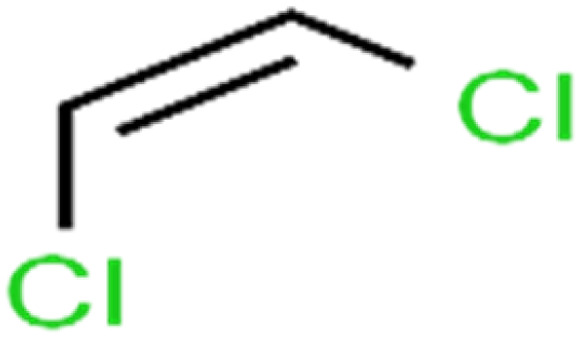	13.4	11	Test
43	*cis*-1,3-Dichloropropene	*cis*-1,3-DCP	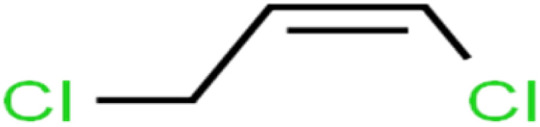	35.8	48	Training
44	Dibromochloromethane	DBCM	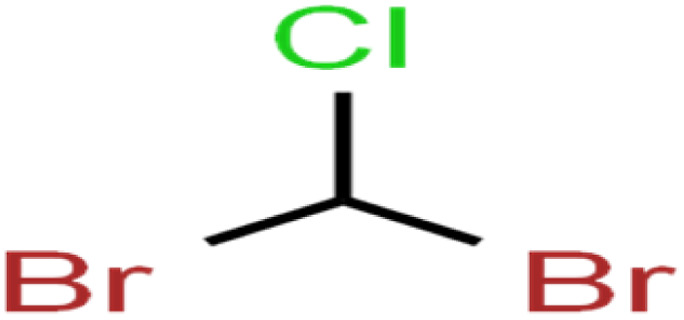	74.2	78	Training
45	Dibromomethane	DBM	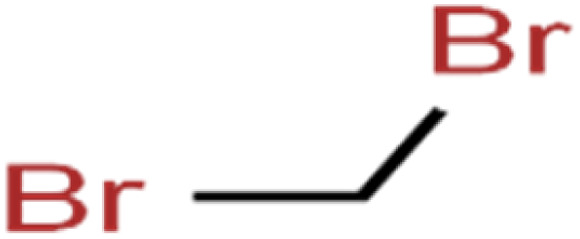	10.9	25	Training
46	Ethylbenzene	EB	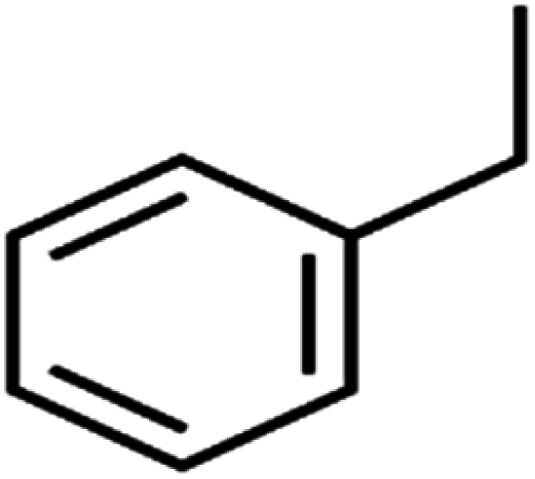	92.1	87	Training
47	Hexachloro-1,3-butadiene	HCBD	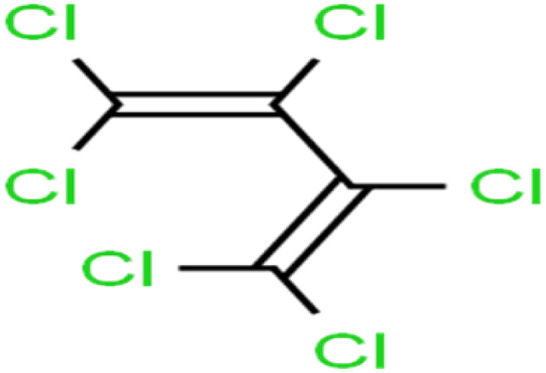	95.7	>96	Validation
48	Isopropyl alcohol	IPA	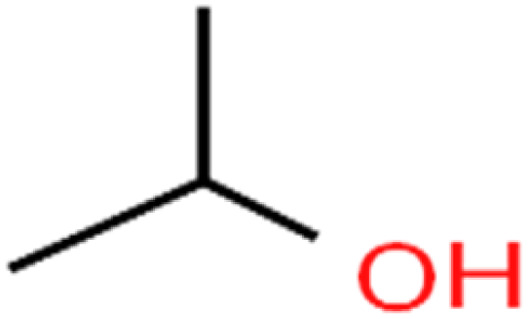	83.6	91	Training
49	Isopropyl benzene	Cumene	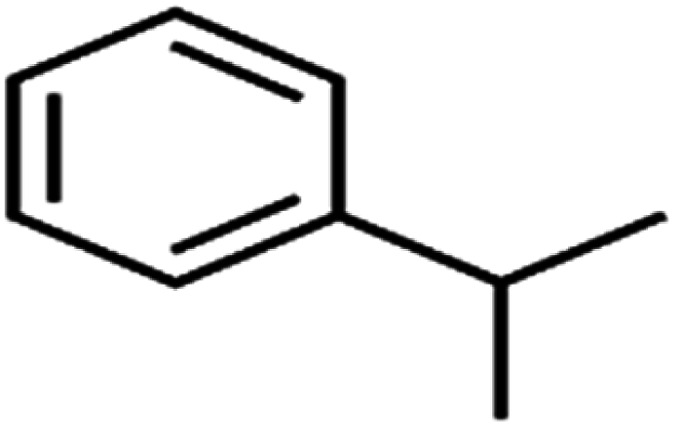	94.7	97	Training
50	Isopropyl ether	IPE	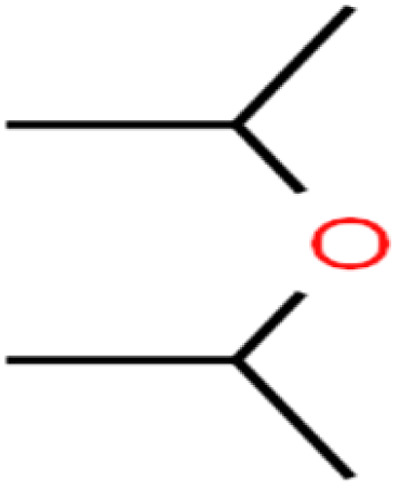	97.3	99	Test
51	Methyl *tert*-butyl ether	MTBE	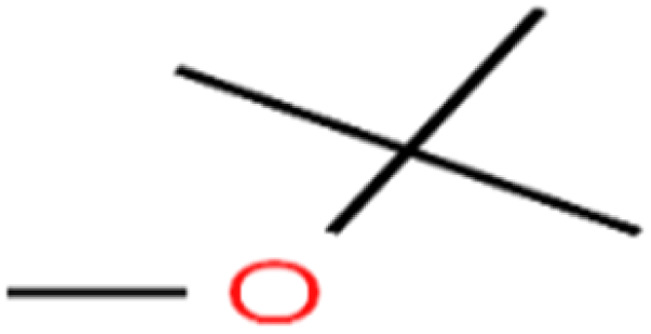	97.6	99	Training
52	Methylene chloride	MC	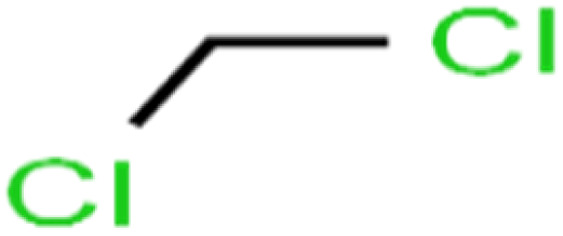	14.8	10	Training
53	*m*-Xylenes	*m*-Xylenes	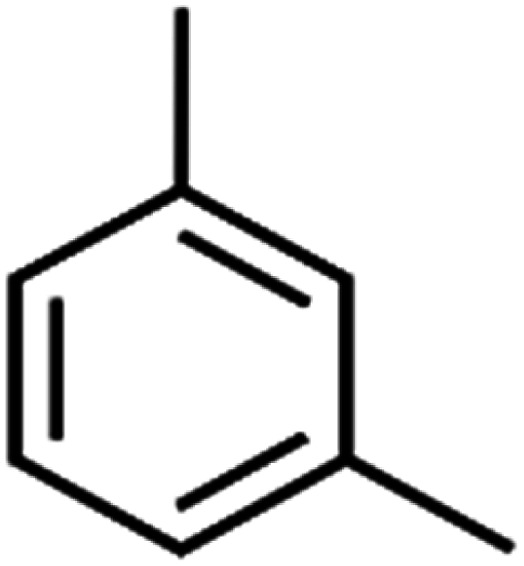	93.9	88	Validation
54	*p*-Xylenes	*p*-Xylenes	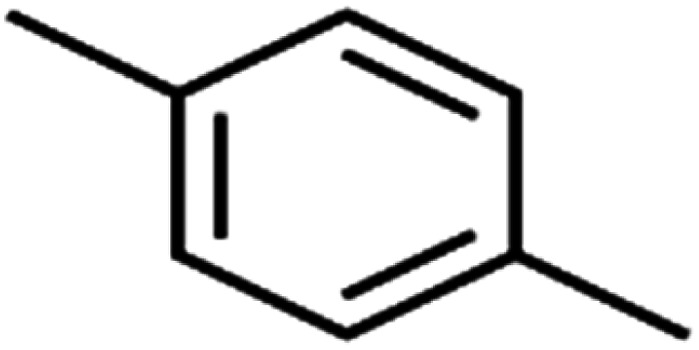	91.3	88	Validation
55	Naphthalene	Naph	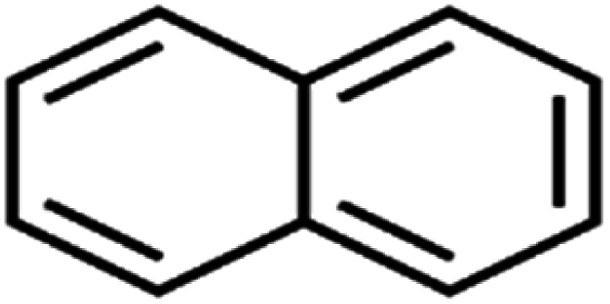	89.0	91	Training
56	*n*-Butylbenzene	*n*-BB	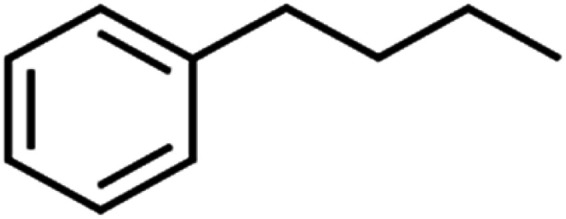	95.8	90	Training
57	*n*-Propylbenzene	*n*-PB	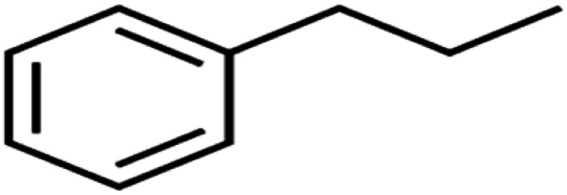	93.2	88	Training
58	*o*-Xylene	*o*-Xylene	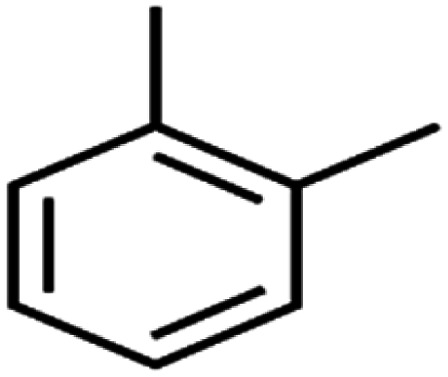	95.5	96	Training
59	*sec*-Butylbenzene	*s*-BB	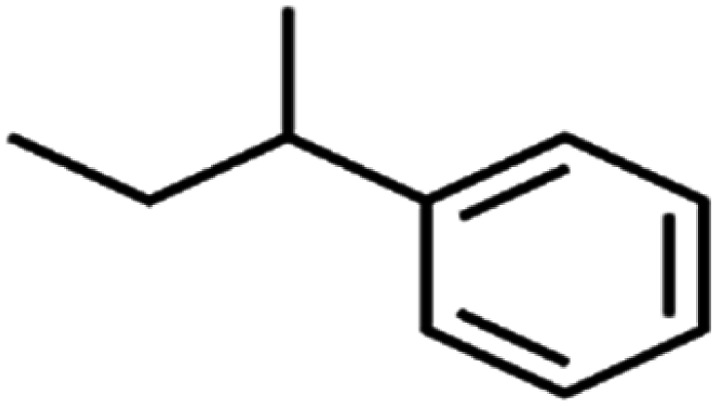	96.4	98	Test
60	*tert*-Amyl methyl ether	TAME	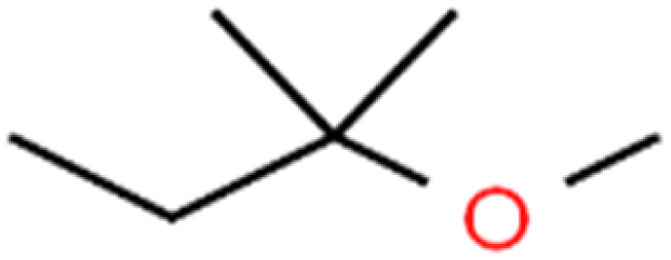	98.8	99	Training
61	*tert*-Butyl alcohol	TBA	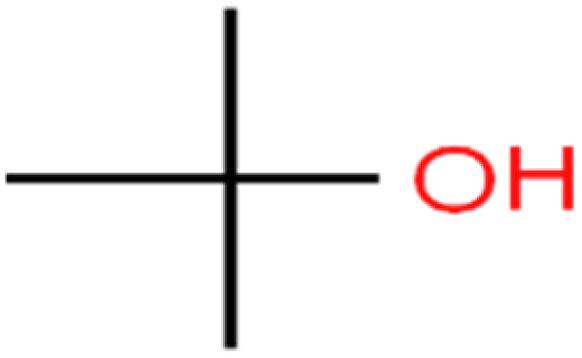	95.6	99	Validation
62	*tert*-Butyl ethyl ether	TBEE	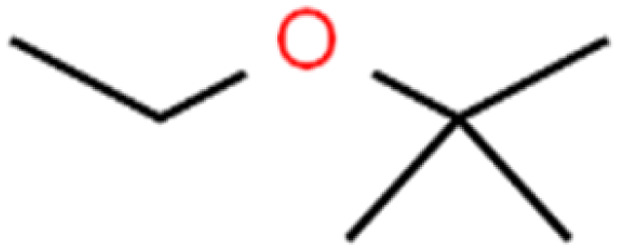	98.6	99	Test
63	*tert*-Butylbenzene	TBB	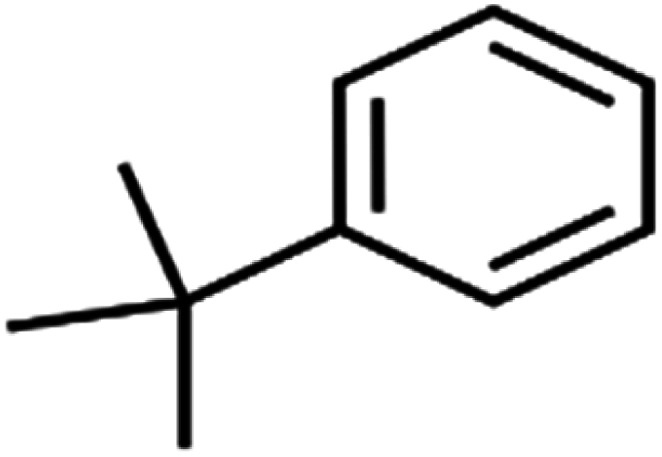	98.0	>96	Training
64	Tetrachloroethene	PCE	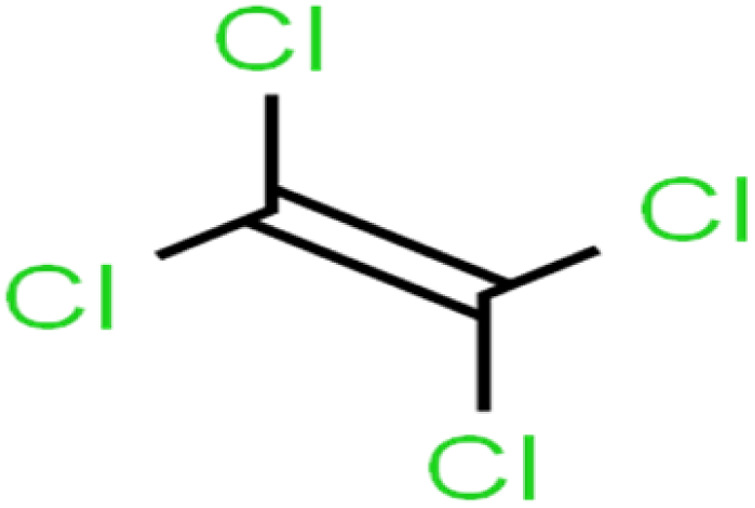	82.0	83	Training
65	Toluene	Toluene	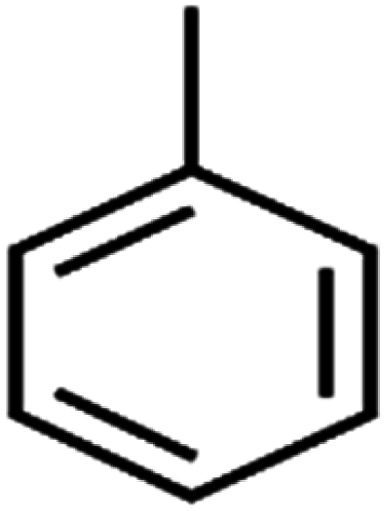	86.0	82	Training
66	*trans*-1,2-Dichloroethene	*t*-1,2-DCE	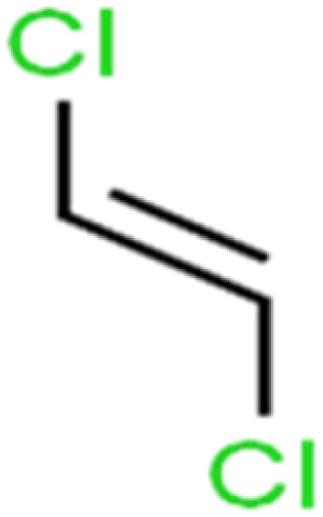	13.4	15	Training
67	*trans*-1,3-Dichloropropene	*t*-1,3-DCP	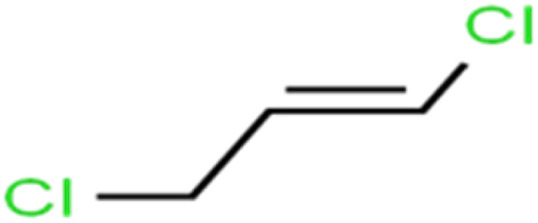	39.2	27	Training
68	*trans*-1,4-Dichloro-2-butene	*t*-1,4-DCB	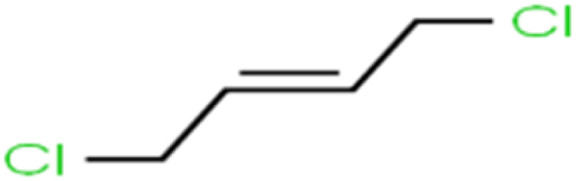	52.3	51	Training
69	Trichloroethene	TCE	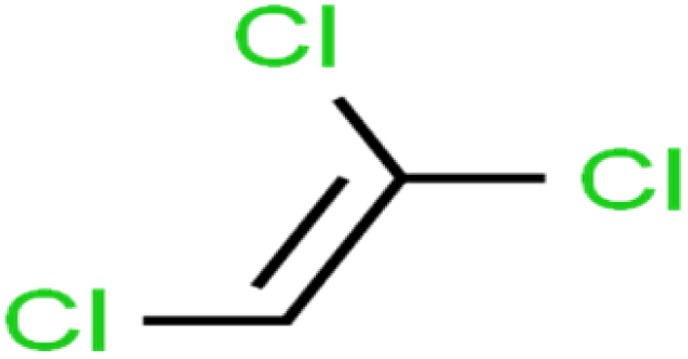	42.8	46	Training
70	Vinyl acetate	VA	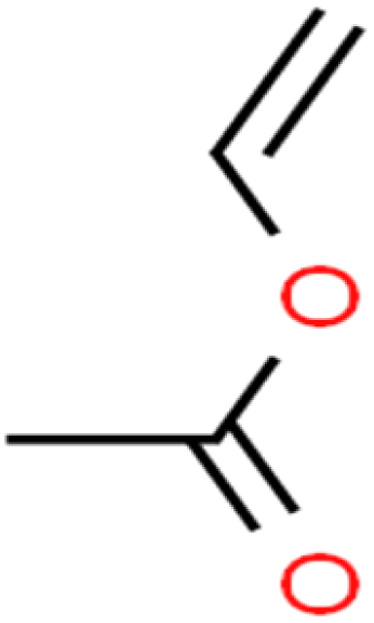	49.3	46	Training
71	Vinyl chloride	VC	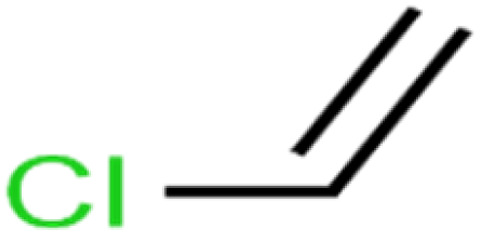	19.4	17	Training
72	Vinylbenzene	Styrene	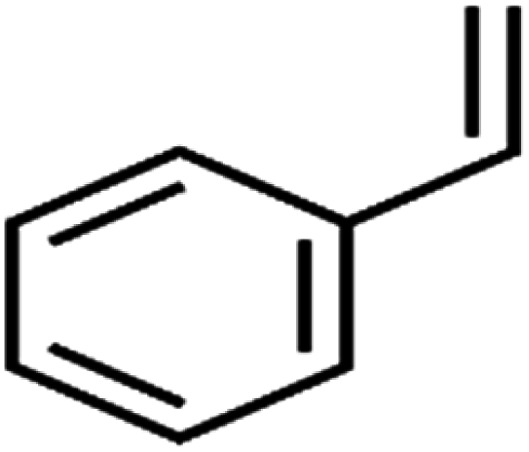	86.6	75	Test

All molecular structures (Table 1) were created by the Gaussview 5.0 program^59^ and optimized in the Gaussian 09 program with the semi-empirical PM6, standing for parameterization method 6.^60^ PM6 is one of the developed semiempirical techniques which is commonly applied for optimizing the structures of molecules.^61^ Dragon 5.5-2007 program was used to calculate the molecular descriptors for each compound.^62^ All statistical computations were conducted in MATLAB 7.0 software and ANN was executed using Matlab Neural Network Toolbox (nntools).^46^

### 2.2 Artificial neural network

An artificial neural network (ANN) is a subset of a machine learning method that is simulated from biological neural systems. ANN includes many artificial neurons or nodes that are interconnected by simple processing units, *i.e.* neurons. A connector node shows artificial synapses. This node exists both among input layers and hidden layers and among the neurons and an output layer, called weight (*W*_*ij*_). The input data is processed in a node as in the following eqn (1):1
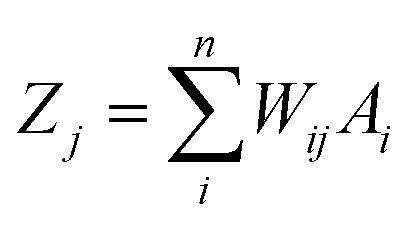
where *Z*_*j*_ is the value of *j*th hidden node and *W*_*ij*_ is the weight connecting the *i*th input node to the *j*th hidden node. *A*_*i*_ is a normalized value of *i*th independent variable and represents the *i*th value of the input node. In the ANN algorithm, input and output data are replaced to a new range of value between −1 to +1 as below in eqn (2):2
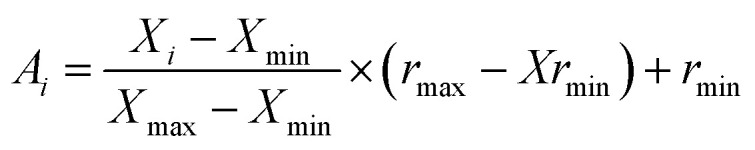
where *i*th an actual variable is *X*_*i*_, the normalized amount of *X*_*i*_ is *A*_*i*_; *X*_min_ is minimum and *X*_max_ is the maximum value of *X*_*i*_. *r*_min_ and *r*_max_ are related to the limits of the range where *X*_*i*_ must be scaled.

One of the most common ANN paradigms used for nonlinear models is the back-propagate feedforward neural network (BPFF), which has been applied in this study.^49,50,53,63^ In the ANN based on the BPFF method, the weights must be changed in each iteration to achieve the smallest difference between the experimental and predicted outputs by the model. Eqn (3) shows the changing weight in each iteration:Δ*W*_*ij*_ + *W*_*ij*_ → *W*_*ij*_

3 Δ*W*_*ij*_ = *η*(*t* − *o*)In_*i*_

where, *t* is the amount of target and *o* is the output value of the network, for each sample, and the value of weight change in each iteration is controlled by *η*, which is called the learning parameter. The amount of *η* is mostly smaller than 0.1 and it reduces and its effectiveness will decrease as the number of iterations increases.

In the BPFF-ANN method, the functioning of the nodes arranged in layers is wherein the input layer receives inputs from the real world. The succeeding layer receives weighted outputs from the preceding layer as its input resulting and, the outputs of the last layer constituting the outputs to the real world.^44,45,52,53^ A node in the hidden or output layer performs two tasks: first, it sums a bias value plus the weighted inputs from numerous connections and next applies a transfer function to the sum. Second, it propagates the resulting value through outgoing connections to the nodes of the succeeding layer where it undergoes the same process. The number of nodes in the input and output layers is revealed by the number of independent and dependent variables, respectively. In this work, the independent variables are the molecular descriptors and the dependent variable is the rejection parameter. The network can learn the relationships between independent and dependent variables by repeatedly comparing the predicted rejection and the experimental rejection; and the subsequent adjustment of the weight matrix and bias vector of each layer by a backpropagation training algorithm.

To perform an ANN analysis, various initialization method is done to decrease the possibility of convergence to a local minimum and the initialization is used with random weights. The data used in this method are randomly classified into three sets: the test set is employed to avoid the overfitting problem and also shows the optimal number of nodes in the hidden layer, the training set is utilized to adjust the parameters of the weights and finally, the validation set is utilized to confirm the real predictive power of the ANN model.^46,54,63–66^

## 3. Result and discussion

This work is aimed at modeling the QSAR data of 72 ECs based on the ANN strategy to predict the rejection of ECs according to their structural properties. The crucial step in the analysis is optimizing the ANN model as described below.

### 3.1 Optimizing ANN model

The first issue in ANN modeling is using a few variables to reduce the complexity of the analysis, prevent overfitting/overtraining and diminish computational time and improve the prediction power for new samples.^41–44,46,67^

In this work, the number of molecular descriptors computed by Dragon software was 3224 and a few important ones should be selected. 1900 out of 3224 descriptors were with all zero element values; therefore, they were omitted from the data set. Furthermore, 980 out of remained descriptors had a high correlation with each other (*R* > 0.90), which means they possessed similar information about the molecules, which were removed from the next consideration.^68^ Finally, based on stepwise regression analysis, 11 out of the remained descriptors from the previous step had a high correlation with response and less correlation with each other. These significant descriptors were ranked based on their *p*-values in ascending order and the four first descriptors were selected for further analysis (the less *p*-value of the parameter is the more probability of the parameter's significance). The selected descriptors were represented in Table 2.

**Table 2:** The selected molecular descriptors for the QSAR method

ID	Name	Description	Block
1	SIC1	Structural information content (neighborhood symmetry of 1-order)	Information indices
2	R2e	R autocorrelation of lag 2/weighted by Sanderson electronegativity	GETAWAY descriptors
3	EEig03d	Eigenvalue 03 from edge adj. matrix weighted by dipole moment	Edge adjacency indices
4	ESpm14u	Spectral moment 14 from edge adj. matrix	Edge adjacency indices

The second issue in ANN analysis is finding the optimal number of hidden layers and their nodes. Here, ANN optimization was conducted using a toolbox in MATLAB (nntools) based on the BPFF algorithm. The main parameters of the network in the toolbox were as follows: the percentage of data amounts in each classified set (testing, training, and validation), topology, training algorithm, and its factors as presented in Table 3.

**Table 3:** Network parameters in the MATLAB software toolbox

Topology	four inputs, one output, and one hidden layer with four neurons (4 × 4 × 1)
Data	Training set: 69.44% randomly selected observation data (50 data values)
	Test set: 15.27% randomly selected observation data (11 data values)
	Validation set: 15.27% randomly selected observation data (11 data values)
Beginning function	Log-sigmoid
Training algorithm	Levenberge–Marquardt
Loss function conditions	Minimum MSE
Stopping conditions	The network stops in one of three ways
	Validation check > 10
	Minimum gradient < 10^−7^
	Momentum speed > 10^10^

To find the optimal nodes in the hidden layer, different models with one hidden layer were constructed in which the nodes varied between 1 and 7. Then, the efficiency of each model was evaluated based on correlation coefficient (*R*^2^), mean square error (MSE), mean absolute percentage error (MAPE), and residual mean squared error (RMSE).

The above parameters are determined as follows:^17,41,42,69,70^4
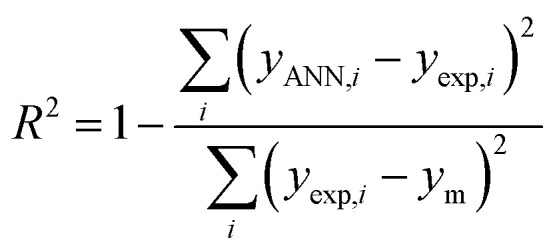
5
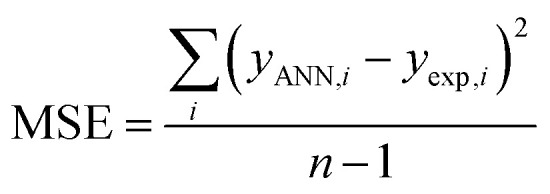
6
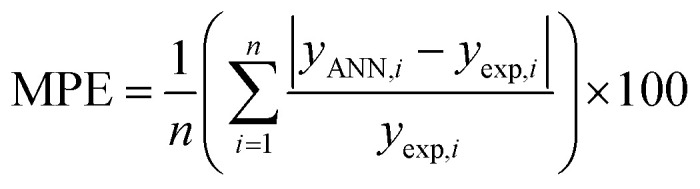
7
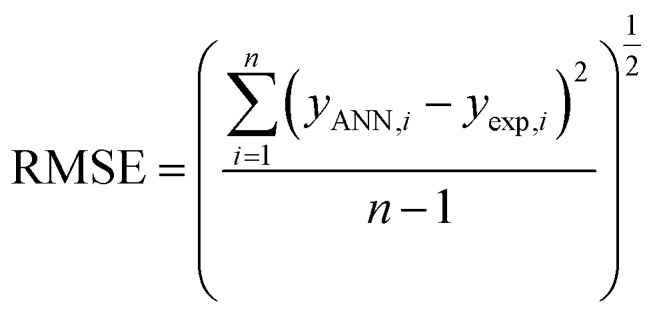
where *y*_exp_,_*i*_ and *y*_ANN_,_*i*_ are the experimental and predicted values of rejection for *i*th molecule with the membrane, and *y*_m_ is the mean of *y*_exp_ in the above equations. And, *n* is the number of compounds in training, test, and validation sets.

The main target is minimizing the MSE error of the test set, as data that is not utilized during the train iterations, confirms the power of ANN's ability in the prediction of the new data set.

**Fig. 1:**
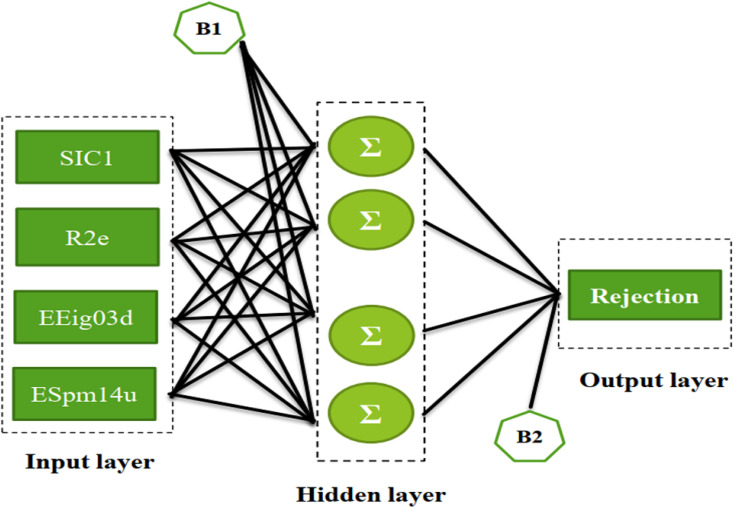
The scatterplots of descriptors (input) *versus* the ANN predicted model (output).

The ANN optimal structure was achieved according to the maximum amount of *R*^2^ and the minimum amount of the MSE of the test set. Fig. 1 displayed a topology of the optimal model in this work.

**Fig. 2:**
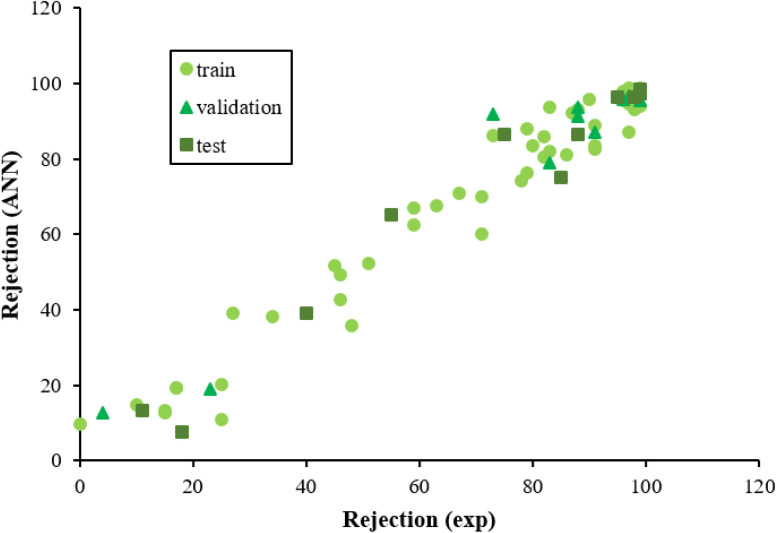
The scatterplot of predicted rejections of molecules by the ANN method *versus* corresponding experimental rejections in different data sets.

The ECs rejection was predicted using the optimized ANN model in three sets test, train, and validation, reported in Table 1. The whole of the obtained results was converted to the initial state and plotted in Fig. 2 against the corresponding experimental rejections.

In ANN analysis, the statistical parameters of *R*^2^, MSE, MPE, and RMSE were obtained for the data sets of the training, testing, and validation, as reported in Table 4. The *R*^2^ amounts between the predicted and experimental results show the ANNs are highly effective for making the relationship between the structural properties of ECs and their rejection.

**Table 4:** Statistical parameters of the ANN and MLR model

Set of data	*R* ^2^		MSE		RMSE		MPE	
	ANN	MLR	ANN	MLR	ANN	MLR	ANN	MLR
Total	0.9528	0.8753	41.2	128.6	6.4	11.3	12.2%	24.3%
Training	0.9434	0.8625	43.6	123.3	6.6	11.1	14.2%	12.4%
Test	0.9583		43.9		6.6		7.3%	
Validation	0.9759	0.9280	28.0	158.0	5.2	12.6	10.0%	46.1%

In the following, the obtained data were investigated by the MLR model,^71^ and the results were evaluated with the ANN algorithm to reveal the necessity of applied nonlinear models in this research. The QSAR linear equation can be written as in the following:

8 *y* = 0.1632 − 0.1963528SIC1 + 0.4113547R2e + 0.1675084EEig03d + 0.867939ESpm14u

SIC1, R2e, EEig03d, and ESpm14u are the same parameters as reported in Table 2. *y* is the rejection of each molecule by the RO membrane.

**Fig. 3:**
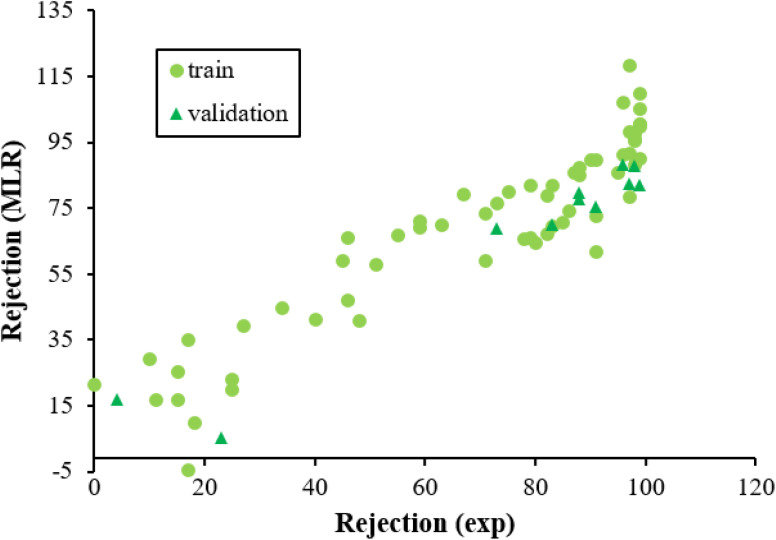
The scatterplot of predicted rejection of molecules by MLR method *versus* experimental rejection of molecules in different data sets.

Fig. 3 displayed the association between the predicted and experimental results of the MLR technique. However, the fit is worse as compared to that given for ANN analysis (Fig. 2), confirming the efficiency of the ANN method for analyzing this QSAR data.

**Fig. 4:**
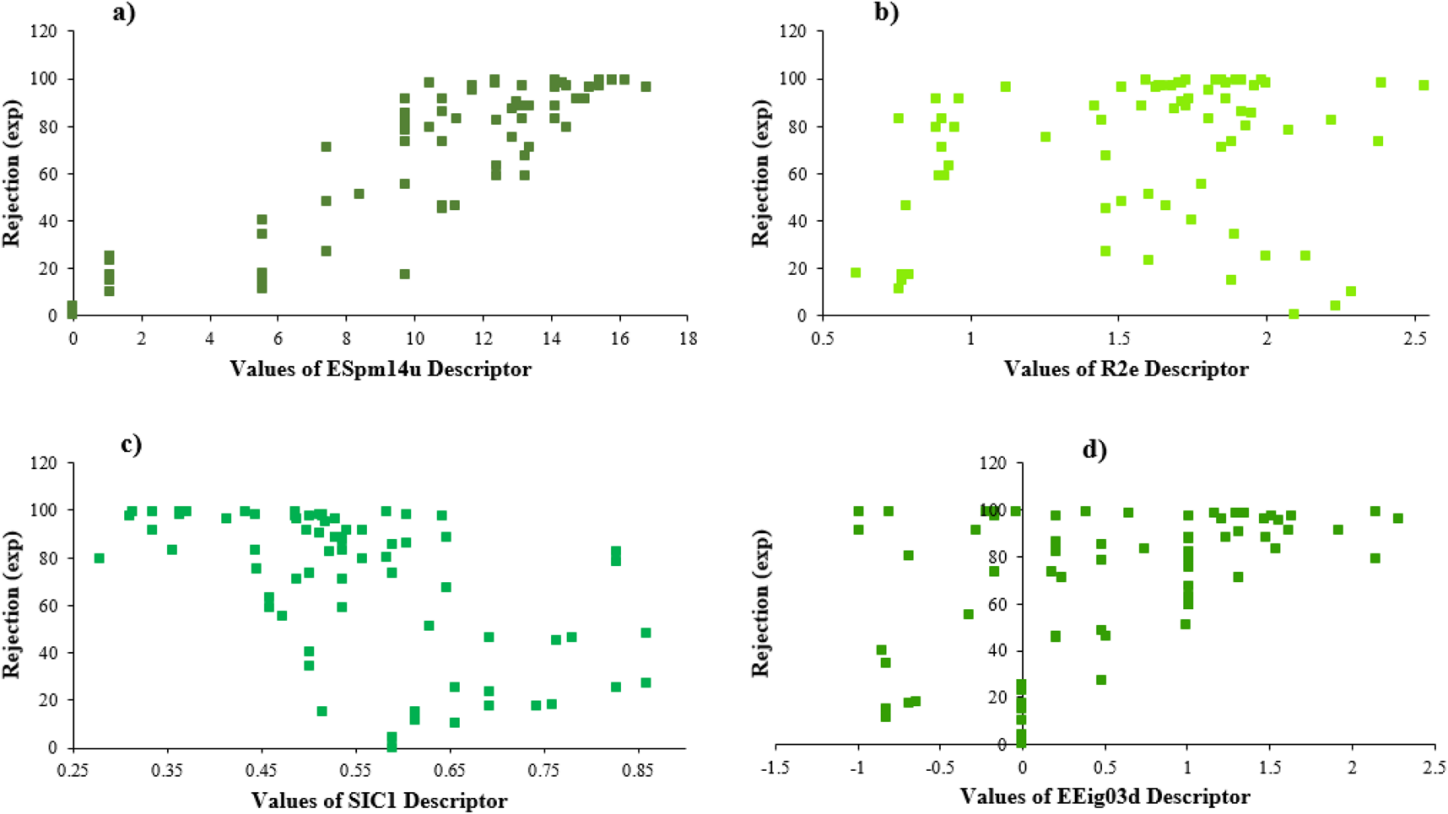
The plots of experimental rejection *versus* the values of each selected descriptor.

Furthermore, Fig. 4 illustrates the scheme of experimental rejections *versus* descriptors SIC1, R2e, EEig03d, and ESpm14u. This figure shows the nonlinear relationship between the structure of molecules and rejections.

### 3.2 Impact of input variables

The weights are numerical parameters in the ANNs algorithm that can be used to calculate the relative importance of each input data on the output target utilizing Garson's algorithm, as follows (9):^72^9
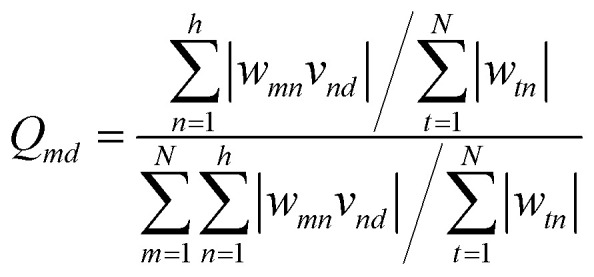
where the value of weight between the *m*_th_ input and the *n*_th_ hidden nodes is *w*_*tn*_ and *v*_*nd*_ shows the weight amount between the *n*_th_ hidden nodes and the *d*_th_ output data.

Based on the Garson we estimate the percentage of the effective input variables on rejection by combining input-hidden and hidden-output connection weights. The results were presented in Table 5. The trend of the importance of input descriptors can be expressed as ESpm14u > R2e > SIC1 > EEig03d. Indeed, the numerical value of the molecular descriptors is important for interpreting the relationship between compound rejection and molecular descriptors and is useful for explaining the results as discussed below.

**Table 5:** Effective weight matrix for the ANN model

Input descriptors				Hidden neurons	Hidden to out
SIC1	R2e	EEig03d	ESpm14u		
−1.0877	1.3881	0.0052	0.9426	H1	0.7219
1.8117	−1.3380	−1.8139	−2.1147	H2	−0.2206
3.6353	−0.7606	−1.5711	3.9892	H3	0.1505
2.2321	−2.9344	2.5124	3.6544	H4	0.5867
24.97	25.72	17.35	31.94	Relative importance (%)	

EEig03d represents the eigenvalue 03 from the edge adjacency matrix weighted by dipole moments. This descriptor was assigned to the polarity of molecules, which mostly explains the electronic effect of the compounds and the hydrophobic properties. On the other hand, molecular polarity is an important parameter in rejection by RO membranes because this factor influenced the interaction of the molecules with the membrane and, in turn, the diffusion of the molecules.^57,73,74^

According to the results, the presence of non-polar or very low polar functional groups increases the numerical value of EEig03d and for compounds with high polarity functional groups, the value is negative. The results showed similar molecules with halogen groups have lower EEig03d than those of methyl groups Table S1.† For instance, the EEig03d value for 2-CT is 1.48 while for CB is 1, as a result, the rejection of 2-CT (88%) is more than CB (63%), and Naph has a 1.61 value of EEig03d by 91% rejection while benzene with 1 value of EEig03d shows 79% rejection.

The methyl group also reduces the polarity, as compounds with one methyl functional group are more polar than those of more methyl functional groups. Hence, these molecules have a low value of EEig03d and are followed by a decrease in rejections. On the contrary, the polar functional groups lead to higher partitioning into the polyamide membrane resulting in lower rejection which coincides with their low value of EEig03d.^57^ The effect of EEig03d on the polarity of compounds and eventually on RO rejections is presented for two pairs of the same molecules in Table S1.† Such examples are the pair of compounds *m*-xylenes and toluene; 4-IPT and cumene; *t*-1,3-DCP and *t*-1,4-DCB; 2-But and 2-Hex; 1,2-DB-3-CP and DBCM; MTBE and TBEE. It should be mentioned in MLR analysis, EEig03d reported positive regression coefficients, which offered the descriptor had a positive effect on rejection, consequently, by increasing the value of EEig03d, rejection is increased.^74^

ESpm14u is the first molecular descriptor with a high positive contribution and displays the spectral moment 14 from the edge adjacency matrix. Spectral moments are the most important factors that can be calculated to many different matrices utilized to represent the structure of the states of various systems. The spectral moments *k* of a matrix *M* of the molecular graph *G* is one of the most suitable molecular descriptors for QSAR models of complex structures.^75,76^ The line graph of the chemical graph represents the sum of all Self-Returning Walks of length *r*, that begins and ends with a similar vertex.^77,78^ In the present study, the results of the MLR model showed that ESpm14u has a positive effect on rejection, which is in good agreement with the high value of ESpm14u for large molecules. Interestingly enough, the numerical value of ESpm14u for larger molecules increases.

There are four parameters attributed to the molecular size as follows: MW, volume, and molecular length or width, all of which are used to explain the rejection of organic compounds by the RO membrane. For example, the ESpm14u value for *t*-1,4-DCB is 8.341, in contrast, for *t*-1,2-DCE is 5.549 because in *t*-1,4-DCB the number of atoms is more than *t*-1,2-DCE, as a result, the rejection of *t*-1,4-DCB (51%) is higher than *t*-1,2-DCE (15%). And TBA has a 15.381 value of ESpm14u by 99% rejection compared to IPA has 9.704 and 91% rejection. Similar examples of the pair of compounds are as follows (Table S1†): 1,1,2,2-TCA and 1,1-DCA; 1,1-DCE and 1,1-DCP; 2-But and 2-Hex; MIBK and acetone; BCM and BDCM; *cis*-1,2-DCE and *cis*-1,3-DCP; DBCM and DBM; EB and cumene.

R2e is one of the types of molecular descriptors obtained from the R indices of the R-GETAWAY group. In general, GETAWAY is an acronym for topology, atomic masses, and geometry assembly.^79^ Indeed, R-GETAWAY molecular descriptors combine the information provided by the molecular influence matrix with geometric interatomic distances in the compound. The R2e is a kind of autocorrelation of lag 2 weighted by atomic Sanderson electronegativities, which encodes geometrical information given by the chemical information from electronegativity.^76,80^

Here, the result shows that compounds with larger R2e numerical values attributed to the compounds with the more electronegative groups and also lower rejection. For example, 1,2-DCP (R2e = 1.861) has a rejection 91%, in contrast, 1,2-DB-3-CP (R2e = 1.675) has a rejection 97%; EDB (R2e = 1.743) by rejection 40% and 1,2-DCA (R2e = 1.89) by rejection 34%; the numerical value of R2e for CF is 2.381 with rejection 73% and the value of R2e for BF is 1.95 with rejection 85%; MC (R2e = 2.282) has rejection 10% and BDCM (R2e = 2.221) has rejection 82%. It should be noted that the presence of an electropositive group in the molecule makes the R2e value reduces and rejection increases. Such examples are the pair of compounds CA and VC; 1,1,2-TCA and 1,2,3-TCP; BCM and DCM; C-Tet and 1,2,3-TCP.

SIC1 is the structural information content of order 1. It represents a general measure of structural complexity and encodes information about atom equivalence. The high value of SIC1 is a sign of relatively branched, large, and polycyclic compounds.^76,81,82^ As seen in Table S1,† the SIC1 values increase regularly in a series of molecules as branching decreases.

**Fig. 5:**
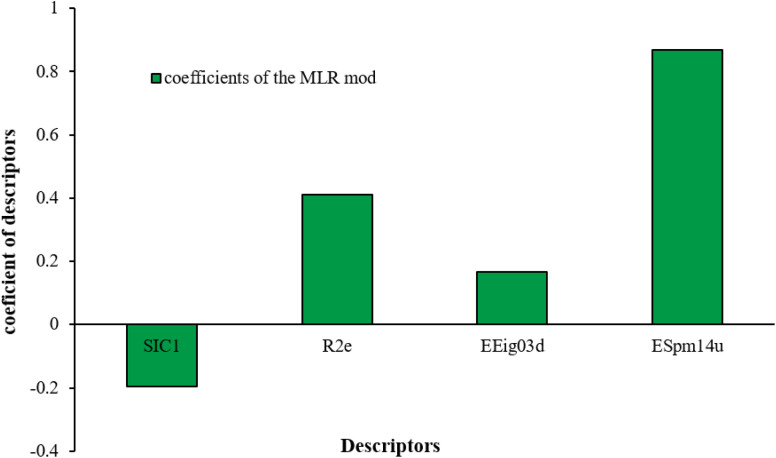
Contribution of all selected descriptors.

The numerical value of SIC1 for 1,1,1,2-TCA is 0.583 and for 1,1,2-TCA is 0.604 as 1,1,1,2-TCA has a higher rejection (Rej = 99%) than 1,1,2-TCA (Rej = 86%), other instance is C-Tet with 0.311 value of SIC1 that has a higher rejection (Rej = 97%) than BF (Rej = 85%) with 0.59 value of SIC1. Similar results are seen in cumene *versus* EB; *n*-BB and *n*-PB; TBB and toluene (Table S1†). From the results of MLR analysis, the negative regression coefficient of SIC1 argues that SIC1 has a negative effect on rejections, which is consistent with the results obtained for the above pair molecules examples. Fig. 5 shows the MLR coefficients *vs.* the descriptors.

## 4. Applicability domain of the developed QSAR models

The scientific validity of a QSAR model is recognized by the Organization for Economic Cooperation and Development (OECD) expert groups, who proposed five principles that should be followed during the construction of QSAR models.^83^ The third principle of OECD is assigned to the applicability domain (AD) for the developed QSAR model. The AD parameter is characterized by the properties of the compounds in the training data set. According to this OECD guideline, only predictions for chemicals falling within the domain of the developed model can be considered reliable, not model extrapolations.^83^

**Fig. 6:**
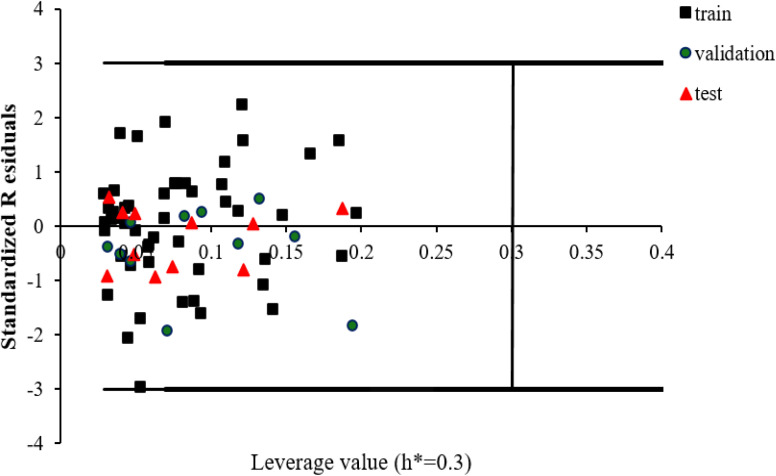
William's plot to visualize AD of the QSAR model.

The leverage approach is one of the most common algorithm to visualize the AD of QSAR models.^84^ In this strategy, the distance of a compound from the centroid of **X**, known as the leverage, is calculated based on the following equation:

10 *h*_*i*_ = **x**_*i*_(**X**^*T*^**X**)^−1^**x**_*i*_^*T*^

where *h*_*i*_ displays the leverage value of *i*th compound, **X** is the descriptors matrix of the training set molecules and **x**_*i*_ is a vector including the descriptors of *i*th molecule (from the training or test set). The critical leverage (h*) can be written as in the following equation:11
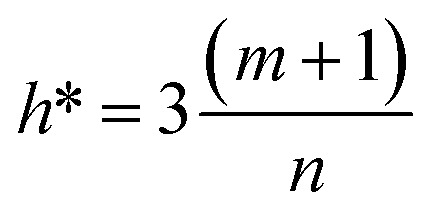
where *n* is the total number of compounds in the training set and *m* is the number of descriptors in the model. William's plot, a plot of standardized residuals *versus* leverage values, is employed to interpret the AD of the model. For an external test compound, the prediction is reliable when its leverage value is less than h* and its standardized residual is no greater than 3 units (±3*σ*). Fig. 6 illustrates William's plot of the QSAR model in this study. As seen, all of the *h*_*i*_ values are within the threshold ±3*σ* and h* = 0.3, a fact which confirms no compounds in the dataset fell outside of the AD as an outlier.

## 5. Conclusions

The present study was aimed at predicting the rejection of emerging contaminants through reverse osmosis by QSAR modeling. The results of the QSAR method were interpreted by two strategies: MLR as a linear model and ANN as a nonlinear modeling approach. During modeling, the four most significant descriptors were identified and selected as listed in the following: ESpm14u, R2e, SIC1, and EEig03d.

The results of QSAR-MLR and QSAR-ANN were compared based on statistical parameters such as *R*^2^, RMSE, and MPE. The lower RMSE and higher *R*^2^ which were obtained by the ANN algorithm (6.4 and 0.9528, respectively) displayed the performance of ANN in detecting the relationship between ECs and their rejections with high predictive power. Moreover, MPEs of the whole data were 12.2% and 24.3% for ANN and MLR, respectively, a fact that confirms the superiority of ANN in predicting the rejection processes of ECs by RO membranes.

## Author contributions

SLM, writing the manuscript, literature review, and model building; SMS, expert view, writing the manuscript, and supervision of the manuscript. All authors contributed to the study's conception and design.

## Conflicts of interest

The authors declare no competing interests.

## Supplementary Material

RA-013-D3RA03177B-s001

RA-013-D3RA03177B-s002
