# Supplementary material for: Predicting rejection of emerging contaminants through RO membrane filtration based on ANN-QSAR modeling approach: trends in molecular descriptors and structures towards rejections
Source: RSC Adv. 2023 Aug 8;13(34):23754–71. doi: 10.1039/d3ra03177b (PMC10407621; doi:10.1039/d3ra03177b)
Supplement: RA-013-D3RA03177B-s002 [file RA-013-D3RA03177B-s002.pdf]

Electronic Supplementary Material (ESI) for RSC Advances.
This journal is © The Royal Society of Chemistry 2023

Predicting Rejection of Emerging Contaminants through RO Membrane Filtration based on ANN- QSAR Modelling Approach: Trends in Molecular Descriptors and Structures towards Rejections

For submission to: **RSC Advances**

By Setare Loh Mousavi, S. Maryam Sajjadi*

[*a]Correspondence Author

S. Maryam Sajjadi:

E-mail: sajjadi@semnan.ac.ir

Tel. (+98) 23 – 31533192

Fax: (+98) 23 33384110

Faculty of Chemistry, Semnan University, Semnan, Iran

Contents:

Table S-1: The relationship between molecular descriptors and structural compounds used in modelling

Table S-1: Molecular descriptors and structural compounds used in QSAR modelling and the experimental and predicted rejection of molecules (associated with %relative error) by ANN analysis

ID	abbrev.	structure	ESpm14u	R2e	SIC1	EEig03d	Rejection (Exp)*	Rejection (ANN)
1	1,1,1,2-TCA	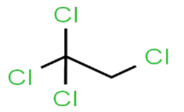	15.777	1.981	0.583	-0.17	99 (±0.9)	94.1
2	1,1,1-TCA	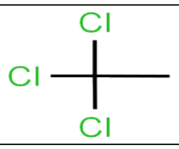	15.381	2	0.604	-0.17	98 (±1.1)	93.1
3	1,1,2,2-TCA	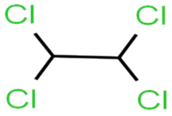	13.17	1.954	0.5	0.2	97 (±1.0)	96.6
4	1,1,2-TCA	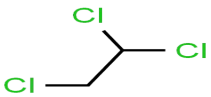	10.852	1.913	0.604	0.2	86 (±2.0)	81.1
5	1,1-DCA	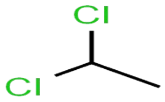	9.704	1.924	0.583	-0.69	80 (±2.0)	83.7
6	1,1-DCE	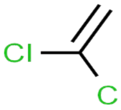	9.704	0.792	0.742	-0.69	17 (±9.0)	19.2
7	1,1-DCP	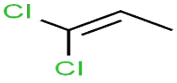	10.852	1.452	0.763	0.2	45 (±2.2)	51.9
8	1,2,3-TCB	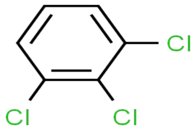	14.972	0.884	0.558	1.92	91 (±3.3)	87.2
9	1,2,3-TCP	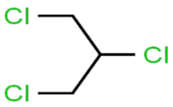	11.685	1.8	0.517	1.26	95 (±1.4)	96.4
10	1,2,4-TCB	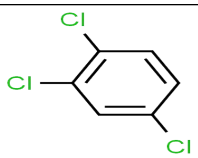	14.473	0.888	0.558	2.13	79 (±6.1)	88.0

ID	abbrev.	structure	ESpm14u	R2e	SIC1	EEig03d	Rejection (Exp)*	Rejection (ANN)
11	1,2,4-TMB	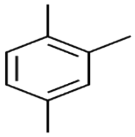	14.473	1.642	0.485	1.63	97 (±1.3)	97.6
12	1,2-DB-3-CP	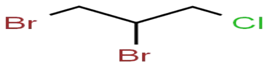	11.685	1.675	0.642	1.50	97 (±1.1)	87.1
13	EDB	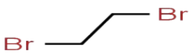	5.549	1.743	0.5	-0.85	40 (±3.9)	39.1
14	1,2-DCB	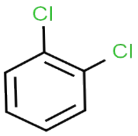	14.084	0.902	0.535	1.53	83 (±5.3)	78.9
15	1,2-DCA	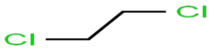	5.549	1.89	0.5	-0.83	34 (±4.1)	38.2
16	1,2-DCP	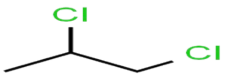	10.852	1.861	0.54	-0.28	91 (±1.6)	82.6
17	1,3,5-TMB	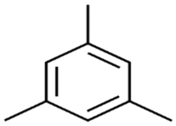	14.094	1.722	0.485	2.14	99 (±0.4)	89.2
18	1,3-DCB	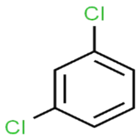	13.393	0.902	0.535	1.31	71 (±7.5)	70.0
19	1,3-DCP	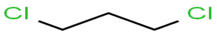	7.431	1.843	0.487	0.24	71 (±2.6)	60.2
20	1,4-DCB	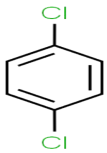	13.23	0.908	0.535	1	59 (±7.9)	62.5
21	1,4-D	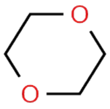	10.397	2.388	0.362	1.34	98 (±0.7)	98.5
22	2-But	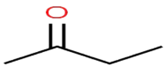	10.852	1.881	0.501	0.17	73 (±2.7)	86.1

ID	abbrev.	structure	ESpm14u	R2e	SIC1	EEig03d	Rejection (Exp)*	Rejection (ANN)
23	2-CT	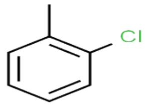	14.084	1.418	0.646	1.48	88 (±4.0)	86.7
24	2-Hex	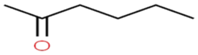	11.259	1.805	0.442	0.73	83 (±2.6)	93.8
25	4-CT	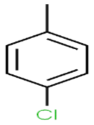	13.23	1.454	0.646	1	67 (±6.9)	71.0
26	4-IPT	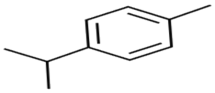	14.328	1.704	0.516	1.29	98 (±0.6)	96.6
27	MIBK	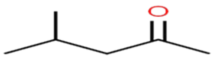	12.358	1.863	0.442	0.63	98 (±0.8)	95.9
28	acetone	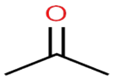	9.704	1.775	0.473	-0.32	55 (±3.8)	65.2
29	Ace-N	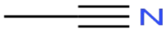	1.099	1.597	0.693	0	23 (±10.6)	19.1
30	Acr-N	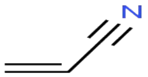	5.549	0.616	0.758	-0.64	18 (±8.1)	7.5
31	benzene	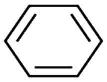	10.397	0.944	0.279	1	79 (±1.9)	76.3
32	BB	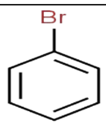	12.41	0.893	0.46	1	59 (±6.9)	67.1
33	BCM	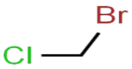	1.099	2.13	0.828	0	25 (±4.1)	20.2
34	BDCM	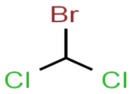	9.704	2.221	0.828	0.2	82 (±2.0)	80.4
35	BF	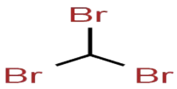	9.704	1.95	0.59	0.48	85 (±3.3)	75.0

ID	abbrev.	structure	ESpm14u	R2e	SIC1	EEig03d	Rejection (Exp)*	Rejection (ANN)
36	BM	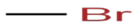	0	2.091	0.59	0	0 (±0.0)	9.7
37	C-Tet	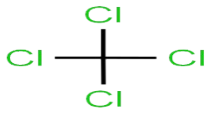	15.381	2.527	0.311	-0.17	97 (-)	98.8
38	CB	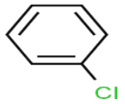	12.41	0.922	0.46	1	63 (±4.8)	67.5
39	CA	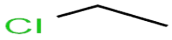	1.099	1.884	0.516	0	15 (±3.6)	12.8
40	CF	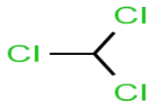	9.704	2.381	0.59	-0.17	73 (±2.4)	91.9
41	CM	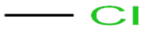	0	2.237	0.59	0	4 (±1.5)	12.8
42	cis-1,2-DCE	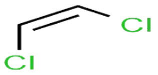	5.549	0.759	0.613	-0.83	11 (±3.8)	13.4
43	cis-1,3-DCP	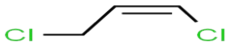	7.431	1.514	0.86	0.47	48 (±2.6)	35.8
44	DBCM	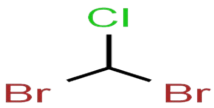	9.704	2.078	0.828	0.48	78 (±3.0)	74.2
45	DBM	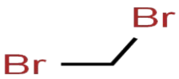	1.099	1.993	0.655	0	25 (±2.7)	10.9
46	EB	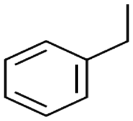	12.869	1.687	0.536	1	87 (±3.9)	92.1
47	HCBD	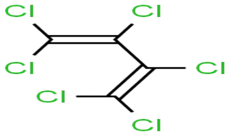	15.148	1.118	0.413	2.28	>96 (±1.6)	95.7
48	IPA	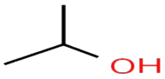	9.704	1.737	0.497	-1	91 (±2.7)	83.6
ID	abbrev.	structure	ESpm14u	R2e	SIC1	EEig03d	Rejection	Rejection

							(Exp)*	(ANN)
49	cumene	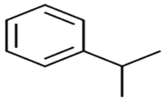	14.087	1.637	0.514	1	97 (±0.9)	94.7
50	IPE	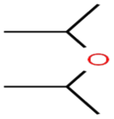	12.358	1.899	0.314	-0.048	99 (±0.7)	97.3
51	MTBE	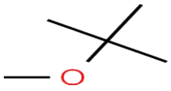	15.777	1.824	0.364	-0.80	99 (±0.5)	97.6
52	MC	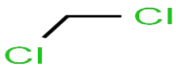	1.099	2.282	0.655	0	10 (±5.0)	14.8
53	m-xylenes	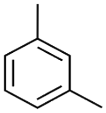	13.393	1.578	0.527	1.22	88 (±3.9)	93.9
54	p-xylenes	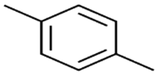	13.23	1.573	0.527	1	88 (±3.9)	91.3
55	Naph	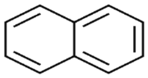	14.73	0.961	0.334	1.61	91 (±3.0)	89.0
56	n-BB	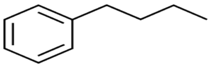	12.996	1.716	0.512	1.30	90 (±3.5)	95.8
57	n-PB	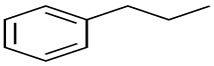	12.969	1.727	0.535	1	88 (±3.5)	93.2
58	o-xylene	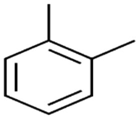	14.084	1.514	0.527	1.46	96 (±1.8)	95.5
59	s-BB	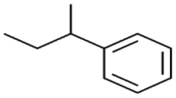	14.301	1.71	0.512	1.15	98 (±0.9)	96.4
60	TAME	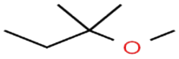	16.127	1.919	0.371	0.38	99 (±0.6)	98.8

ID	abbrev.	Structure	ESpm14u	R2e	SIC1	EEig03d	Rejection (Exp)*	Rejection (ANN)
61	TBA	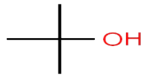	15.381	1.592	0.432	-1	99 (±0.3)	95.6
62	TBEE	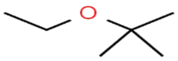	15.824	1.851	0.335	-0.21	99 (±0.4)	98.6
63	TBB	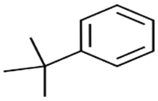	16.758	1.623	0.487	1.20	>96 (±1.4)	98.0
64	PCE	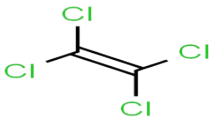	13.17	0.754	0.355	0.2	83 (±5.5)	82.0
65	toluene	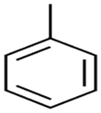	12.41	1.441	0.523	1	82 (±3.8)	86.0
66	t-1,2-DCE	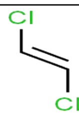	5.549	0.766	0.613	-0.83	15 (±5.3)	13.4
67	t-1,3-DCP	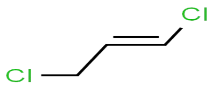	7.431	1.453	0.86	0.47	27 (±3.8)	39.2
68	t-1,4-DCB	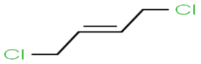	8.384	1.599	0.628	0.98	51 (±4.0)	52.3
69	TCE	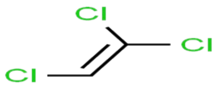	10.852	0.786	0.693	0.2	46 (±2.9)	42.8
70	VA	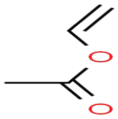	11.149	1.663	0.779	0.51	46 (±10.8)	49.3
71	VC	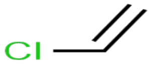	1.099	0.764	0.693	0	17 (±3.9)	19.4
72	styrene	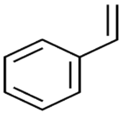	12.869	1.259	0.444	1	75 (±5.7)	86.6

*The standard deviation of the experimental rejection data has been reported in the parenthesis.
